# Global Trends in the Incidence, Prevalence, and Years Lived With Disability of Parkinson's Disease in 204 Countries/Territories From 1990 to 2019

**DOI:** 10.3389/fpubh.2021.776847

**Published:** 2021-12-07

**Authors:** Zejin Ou, Jing Pan, Shihao Tang, Danping Duan, Danfeng Yu, Huiqi Nong, Zhi Wang

**Affiliations:** ^1^Department of Central Laboratory, Guangzhou Twelfth People's Hospital, Guangzhou, China; ^2^Key Laboratory of Occupational Environment and Health, Guangzhou Twelfth People's Hospital, Guangzhou, China; ^3^Guangzhou Occupational Disease Prevention and Treatment Hospital, Guangzhou, China; ^4^Department of Medical Intensive Care Unit (MICU), Guangdong Women and Children Hospital, Guangzhou, China

**Keywords:** Parkinson's disease, global burden of disease, estimated annual percentage change, age-standardized rate, years lived with disability

## Abstract

**Background:** Parkinson's disease (PD) is an increasing challenge to public health. Tracking the temporal trends of PD burden would inform health strategies.

**Methods:** Data of PD burden was obtained from the Global Burden of Disease 2019. Trends in the incidence, prevalence, and years lived with disability (YLDs) of PD were estimated using the annual percentage change (EAPC) and age-standardized rate (ASR) from 1990 to 2019. The EAPCs were calculated with ASR through a linear regression model.

**Results:** The overall ASR of the incidence, prevalence, and YLDs of PD increased from 1990 to 2019, and their EAPCs were 0.61 (95% confidence interval [CI]: 0.58–0.65), 0.52 (95% CI: 0.43–0.61), and 0.53 (95% CI: 0.44–0.62). The largest number of PD patients was seen in the groups aged more than 65 years, and the percentage rapidly increased in the population aged more than 80 years. Upward trends in the ASR of PD were observed in most settings over the past 30 years. Incident trends of ASR increased pronouncedly in the United States of America and Norway, in which the respective EAPCs were 2.87 (95% CI: 2.35–3.38) and 2.14 (95% CI: 2.00–2.29). Additionally, the largest increasing trends for prevalence and YLDs were seen in Norway, with the respective EAPCs of 2.63 (95% CI: 2.43–2.83) and 2.61 (95% CI: 2.41–2.80). However, decreasing trends in PD appeared in about 30 countries, particularly Italy and the Republic of Moldova.

**Conclusions:** Increasing trends in the burden of PD were observed globally, and in most regions and countries from 1990 to 2019. Our findings suggested that the control and management of PD should be strengthened, especially when considering the aging tendency of the population.

## Introduction

Parkinson's disease (PD) is the second most common neurodegenerative disorder (observed only less than Alzheimer's disease), which is characterized by progressive motor symptoms over time (1). In recent years, PD has undergone the fastest growth in prevalence and disability among neurological disorders, and it has become one of the leading causes of disability worldwide (2).

The Global Burden of Disease (GBD) study reported that incident cases of PD were 1.02 million in 2017 (3). There were 6.1 million PD patients reported in 2016 globally, and the age-standardized rate (ASR) of prevalence increased by 21.7% from 1990 to 2016 (4). Years lived with disability (YLDs) is an index measuring the average lifespan of incident cases until rehabilitation or death, and the disability due to that status. YLDs is a widely used index evaluating the health loss caused by PD. Age-standardized rates of YLDs caused by PD increased pronouncedly at 8.9% from 1990 to 2007, and it increased 1.0% during 2007–2017 (3). Studies predicted that the burden of PD would grow substantially in future decades (5, 6). For example, it was estimated that there would be 4.94 million PD patients in China by 2030, which would account for half of the total PD patients worldwide (7). The rapid development of PD has placed a substantial burden on society, individuals, and health system (8–10). Especially, the disability caused by PD dramatically reduced individual health-related quality of life (HRQL), and placed substantial expenses to health system, including drug, rehabilitation, and caregiving (11, 12). However, welfare and health systems in many countries were not prepared for the problems brought by the population aging (13).

Rapid changes in the PD burden have emphasized the necessity for tracking the changing trends in a timely manner, which could inform the development of health strategies. The GBD studies had comprehensively assessed the burden of diseases, injuries, and risk factors over time, which has provided an opportunity to track the temporal trends of diseases. Therefore, this study aimed to estimate the trends of the PD burden at the national, regional, and global levels from 1990 to 2019 using the updated GBD data.

## Materials and Methods

### Data Source

The data on PD was analyzed using the Global Health Data Exchange (GHDx) query tool (http://ghdx.healthdata.org/gbd-results-tool). According to the GBD instructions, data on the PD burden, including the incidence, prevalence, and YLDs, were extracted for sexes, age groups, regions, and countries/territories from 1990 to 2019. A comprehensive overview of the PD burden was presented at the global, regional, and national levels, covering 21 geographic regions and 204 countries/territories. The sociodemographic index (SDI) is a compound index that indicates the association between social development and health outcomes. These regions and countries were classified into SDI quintiles, including low, low-middle, middle, high-middle, and high. Human Development Index (HDI) is a measurement system for evaluating the levels of social and economic development and individual human development. It was downloaded from the United Nations Development Program (http://hdr.undp.org/en/data).

### Statistical Analysis

The estimated annual percentage change (EAPC) and ASR facilitated the horizontal comparison of the changing trends of disease burden among countries, and vertically compared over a long-term interval, which are commonly used indexes in public health research (14, 15). Age standardization is necessary when the data exist for different age structures in multiple populations over time. The ASR (per 100,000 population) was calculated as follows:


$$\begin{array}{l} \text{ASR}=\frac{\sum_{i=1}^{A}\alpha_{i}w_{i}}{\sum_{i=1}^{A}w_{i}}\times 100,000 \end{array}$$

In the formula, *a*_*i*_ corresponds to the age-specific rate in the *i*^th^ age group; *w* corresponds to the number of people in the corresponding *i*^th^ age group among the standard population; *A* corresponds to the number of age groups.

EAPC is a widely used index that describes the trend of ASR, and it was calculated as following. First, the natural logarithm of the ASR was estimated to be linearly regressed with time, and the formula was as follows:


$$\begin{array}{r} \text{y}=\text{α}+\text{βx}+\text{ε}\\ \text{EAPC}=100\times \left[\text{exp}\left(\text{β}\right)-1\right] \end{array}$$

In where y was equal to the natural logarithm of the ASR, and x corresponded to the calendar year. The EAPC and its 95% confidence interval (CI) were estimated using a linear regression model. The judgments of trends were the follows: (1) an increasing trend of ASR was found when both the EAPC value and its 95% CI > 0; (2) a decreasing trend of ASR was found when both the EAPC value and 95% CI <0; and (3) any other trends meant the ASR was stable over time. The associations were evaluated between SDI and ASRs in 2019 among regions to find the potential influential factors of ASR. Furthermore, the ASR in 1990 represented the baseline level of disease burden, and the HDI in 2019 could reflect the level and accessibility of health sources in various countries. Therefore, aim to explore the influential factors of EAPC, a Pearson correlation analysis was used to estimate the associations between EAPC and ASR in 1990, and HDI in 2019, respectively. Collation and analysis of data were conducted using R version 3.6.2 (Institute for Statistical Computing, Vienna, Austria). A *p* < 0.05 was considered to be statistically significant.

## Results

### Trends in the PD Incidence

**Figure 1:**
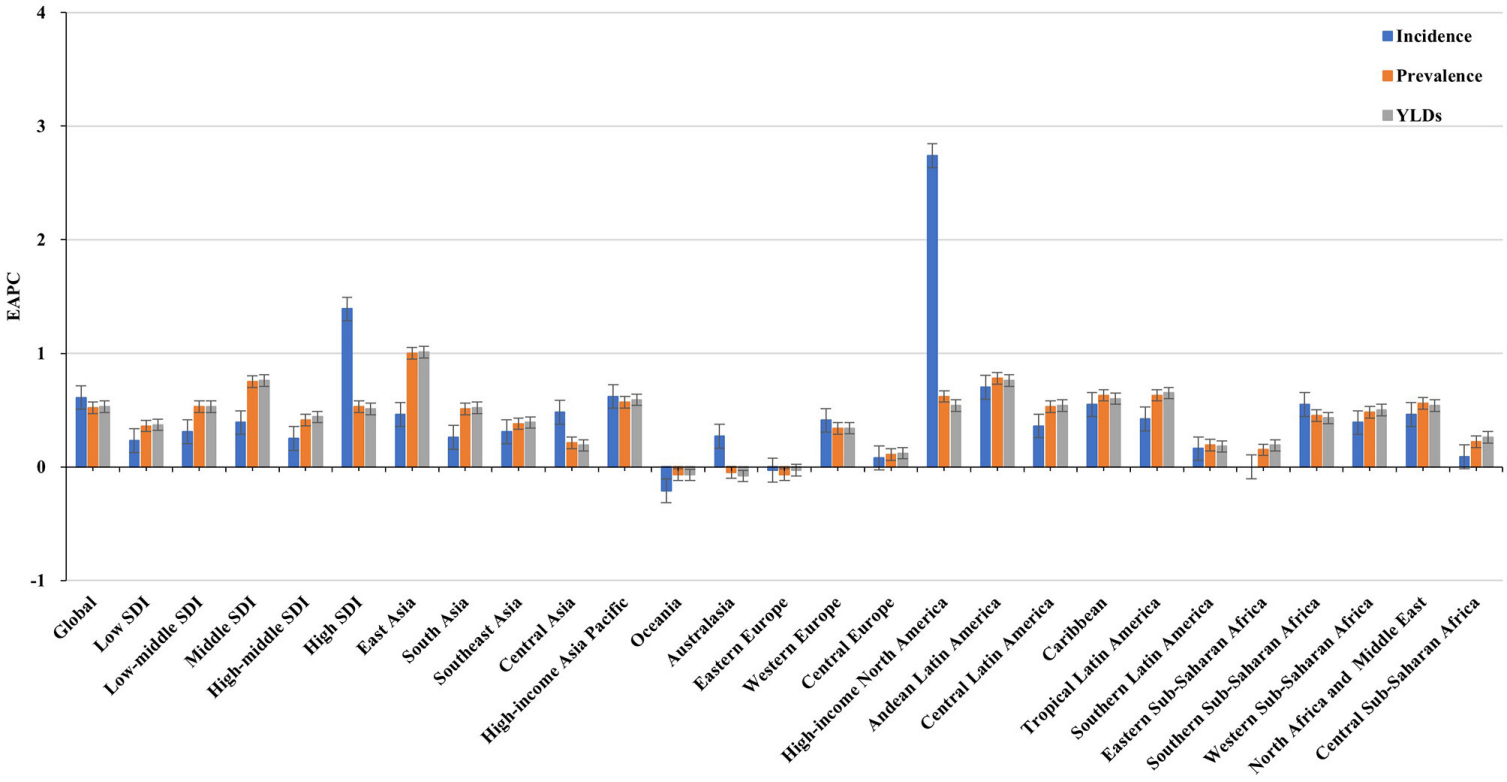
Trends in the ASR of incidence, prevalence, and YLDs of Parkinson's disease in global, SDI areas and geographic regions from 1990 to 2019. ASR, age-standardized rate; SDI, sociodemographic index; YLDs, years lived with disability.

**Figure 2:**
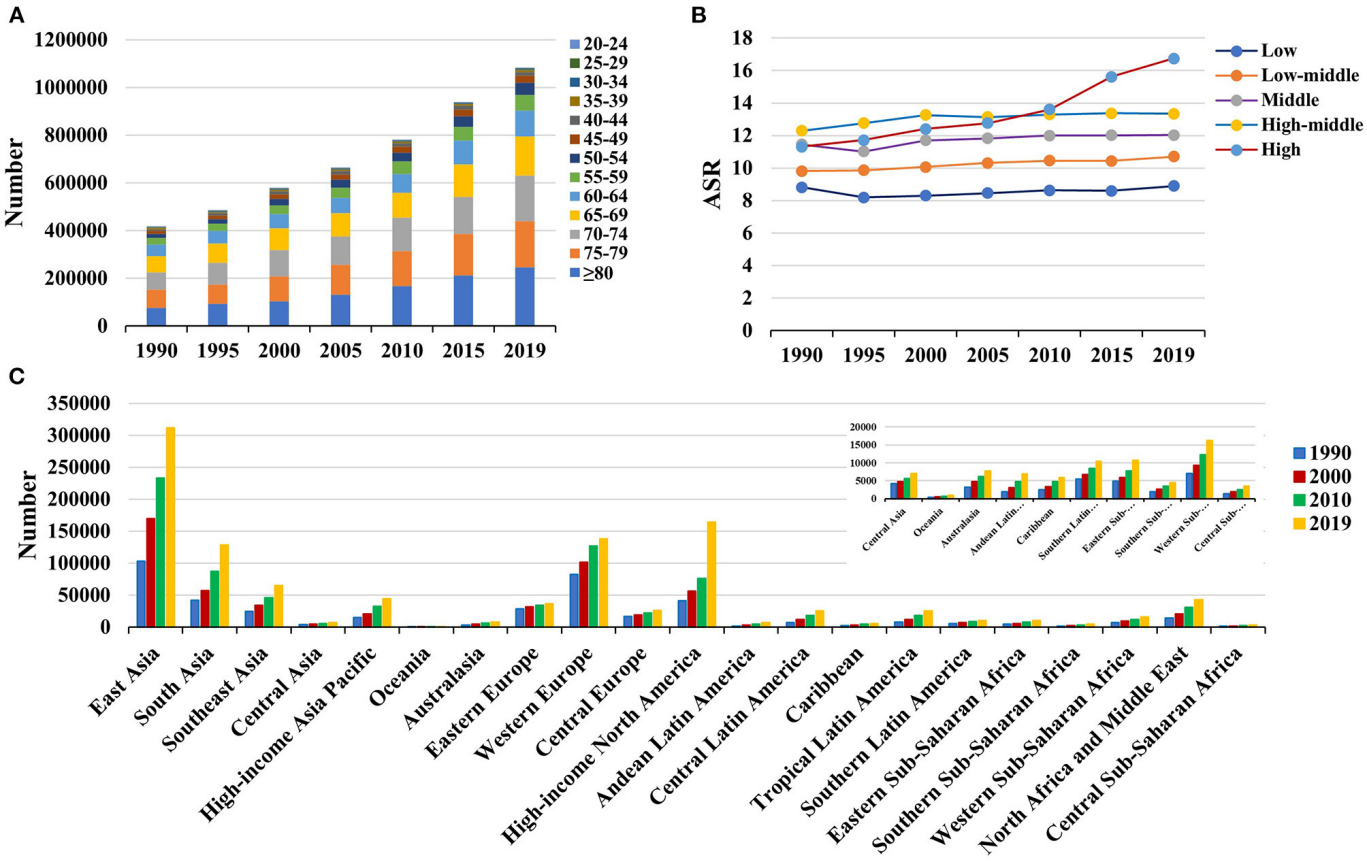
The distribution of Parkinson's disease incidence in age groups, SDI areas and geographic regions from 1990 to 2019. **(A)** was the incident number in age groups; **(B)** was the ASIR in SDI areas; **(C)** was the incident number in geographical regions. ASIR, age-standardized incidence rate; SDI, sociodemographic index.

Globally, the incident number of PD was 1,081.72 × 10^3^ (95% uncertainty interval [UI]: (953.26 × 10^3^-1,211.20 × 10^3^) in 2019, which increased 159.73% since 1990. The overall age-standardized incidence rate (ASIR) was 13.43/100,000 in 2019, and it increased with an annual average of 0.61% from 1990 to 2019 (EAPC = 0.61, 95% CI: 0.58–0.65) (Table 1 and Figure 1). Compared to female patients, male patients had a larger incident number, and a higher increasing trend in ASIR (EAPC = 0.80, 95% CI: 0.75–0.85). Among the age groups, the high incident numbers of PD were observed in the patients aged over 65 years, and the largest increasing percentage occurred in the age group of over 80 years (221.67%) (Supplementary Table 1 and Figure 2A).

**Table 1:** The changes in incidence and prevalence of Parkinson's disease worldwide, and in sexes, SDI areas, and regions, 1990–2019.

**Characteristics**	**Incidence**				**Prevalence**			
	**2019**		**1990–2019**		**2019**		**1990–2019**	
	**Number** **×10^**3**^ (95% UI)**	**ASR/100,000** **(95% UI)**	**Percentage change** **(%)**	**EAPC** **(95%CI)**	**Number** **×10^**3**^ (95% UI)**	**ASR/100,000** **(95% UI)**	**Percentage change** **(%)**	**EAPC** **(95%CI)**
Overall	1,081.72 (953.26–1,211.2)	13.43 (11.84–15.02)	159.73	0.61 (0.58–0.65)	8,511.02 (7,288.53–9,841.38)	106.28 (91.20–122.21)	155.50	0.52 (0.43–0.61)
**Sex**								
Male	645.77 (567.25–720.2)	17.79 (15.75–19.70)	189.19	0.80 (0.75–0.85)	4,667.34 (4,000.97–5,418.91)	129.33 (111.27–149.39)	176.23	0.64 (0.53–0.76)
Female	435.95 (384.17–489.89)	9.95 (8.77–11.18)	125.68	0.26 (0.23–0.29)	3,843.68 (3,306.71–4,441.97)	87.59 (75.33–101.20)	134.16	0.32 (0.25–0.38)
**SDI**								
Low	39.1 1 (34.55–43.86)	8.88 (7.89–9.89)	130.82	0.23 (0.17–0.30)	291.23 (247.53–346.23)	65.71 (55.85–76.84)	149.58	0.36 (0.32–0.40)
Low-middle	134.08 (116.75–152.15)	10.70 (9.31–12.09)	170.86	0.31 (0.29–0.34)	1,026.71 (868.49–1,206.19)	82.16 (69.37–95.93)	188.56	0.53 (0.48–0.57)
Middle	282.09 (241.99–321.83)	12.03 (10.40–13.71)	174.51	0.39 (0.30–0.47)	2,607.78 (2,194.91–3,088.62)	112.30 (94.86–132.34)	227.29	0.75 (0.60–0.90)
High-middle	271.99 (236.23–308.82)	13.34 (11.62–15.10)	116.06	0.25 (0.20–0.30)	2,399.77 (2,036.17–2,803.89)	117.72 (99.88–137.49)	133.19	0.41 (0.33–0.49)
High	330.61 (297.95–362.34)	16.75 (15.11–18.31)	172.85	1.39 (1.22–1.55)	2,181.46 (1,933.30–2,431.20)	108.36 (96.01–120.94)	111.58	0.53 (0.44–0.61)
**Regions**								
East Asia	311.86 (260.77–362.95)	15.26 (12.88–17.70)	202.22	0.46 (0.31–0.62)	2,940.41 (2,419.16–3,533.19)	145.44 (120.83–172.84)	256.35	1.00 (0.74–1.26)
South Asia	128.49 (108.4–149.54)	10.07 (8.50–11.60)	204.06	0.26 (0.23–0.30)	930.14 (770.23–1,111.81)	72.7 (60.27–86.01)	216.46	0.51 (0.46–0.55)
Southeast Asia	65.11 (57.55–73.06)	11.79 (10.52–13.17)	164.21	0.31 (0.25–0.36)	533.59 (455.45–625.12)	99.21 (84.37–115.95)	174.08	0.38 (0.30–0.45)
Central Asia	7.08 (6.33–7.88)	11.40 (10.47–12.42)	67.93	0.48 (0.44–0.51)	50.49 (42.73–59.01)	83.07 (70.59–96.45)	57.39	0.21 (0.17–0.25)
High-income Asia Pacific	44.12 (37.49–50.80)	9.32 (7.97–10.61)	197.14	0.62 (0.53–0.71)	377.83 (314.24–442.54)	78.01 (65.79–91.42)	212.14	0.57 (0.46–0.69)
Oceania	0.87 (0.76–0.98)	14.85 (13.33–16.54)	120.66	−0.21 (−0.25–−0.18)	6.36 (5.24–7.56)	110.76 (91.58–130.67)	129.26	−0.07 (−0.12–−0.03)
Australasia	7.69 (6.78–8.75)	15.23 (13.33–17.39)	136.18	0.27 (0.09–0.45)	70.66 (57.81–85.09)	136.76 (111.78–164.02)	130.12	−0.05 (−0.32–0.22)
Eastern Europe	36.23 (30.44–42.27)	10.30 (8.70–11.96)	27.51	−0.03 (−0.09–0.02)	298.03 (247.35–354.87)	84.41 (70.31–100.50)	27.54	−0.07 (−0.17–0.04)
Western Europe	138.25 (123.84–150.58)	14.61 (13.03–16.05)	68.12	0.41 (0.32–0.50)	1,241.56 (1,062.78–1,419.24)	126.01 (108.86–143.81)	70.22	0.34 (0.24–0.43)
Central Europe	26.31 (24.21–28.47)	11.72 (10.83–12.62)	58.52	0.08 (0.05–0.10)	214.3 (189.41–240.94)	94.09 (83.14–105.87)	65.67	0.11 (0.06–0.15)
High-income North America	164.04 (142.42–186.99)	25.14 (21.88–28.66)	296.11	2.74 (2.30–3.18)	705.49 (638.05–772.01)	107.74 (97.5–117.79)	107.06	0.62 (0.44–0.80)
Andean Latin America	6.84 (6.24–7.44)	12.58 (11.5–13.68)	250.83	0.70 (0.65–0.76)	54.79 (47.10–62.57)	100.80 (86.65–115.25)	257.50	0.78 (0.71–0.86)
Central Latin America	25.32 (22.67–27.96)	11.11 (9.95–12.26)	239.68	0.36 (0.31–0.42)	204.66 (176.47–236.97)	89.95 (77.34–104.04)	259.00	0.53 (0.45–0.61)
Caribbean	5.94 (5.41–6.48)	11.51 (10.51–12.54)	141.84	0.55 (0.47–0.63)	47.32 (41.07–54.2)	91.76 (79.56–105.17)	148.99	0.63 (0.54–0.72)
Tropical Latin America	25.47 (21.5–29.59)	10.83 (9.13–12.58)	223.24	0.42 (0.34–0.49)	205.31 (170.89–242.97)	87.52 (72.56–103.76)	241.68	0.63 (0.54–0.73)
Southern Latin America	10.43 (9.42–11.48)	12.21 (11.03–13.45)	89.97	0.16 (0.09–0.23)	75.58 (64.25–89.71)	88.4 (75.03–104.92)	91.60	0.19 (0.09–0.29)
Eastern Sub-Saharan Africa	10.66 (9.42–12.06)	7.70 (6.85–8.61)	120.32	0 (−0.02–0.02)	77.83 (65.91–93.19)	55.94 (47.39–66.48)	128.53	0.15 (0.12–0.18)
Southern Sub-Saharan Africa	4.53 (3.88–5.22)	9.24 (7.94–10.58)	135.35	0.55 (0.49–0.60)	32.23 (27.13–37.90)	65.31 (54.60–77.13)	134.51	0.45 (0.38–0.53)
Western Sub- Saharan Africa	16.2 (14.54–17.99)	10.76 (9.67–11.83)	132.24	0.39 (0.32–0.46)	109.10 (93.27–127.78)	71.79 (61.18–82.80)	139.86	0.48 (0.37–0.60)
North Africa and Middle East	42.8 (38.33–47.34)	11.40 (10.28–12.54)	192.50	0.46 (0.43–0.48)	309.89 (264.96–362.77)	82.58 (70.23–95.58)	199.48	0.56 (0.52–0.60)
Central Sub- Saharan Africa	3.48 (3.02–3.97)	7.96 (7.05–8.95)	146.44	0.09 (0.05–0.14)	25.44 (20.72–30.86)	58.32 (47.56–69.88)	153.13	0.22 (0.16–0.29)

*EAPC, estimated annual percentage change; ASR, age-standardized rate; CI, confidence interval; UI:, uncertainty interval; SDI, socio-demographic index*.

**Figure 3:**
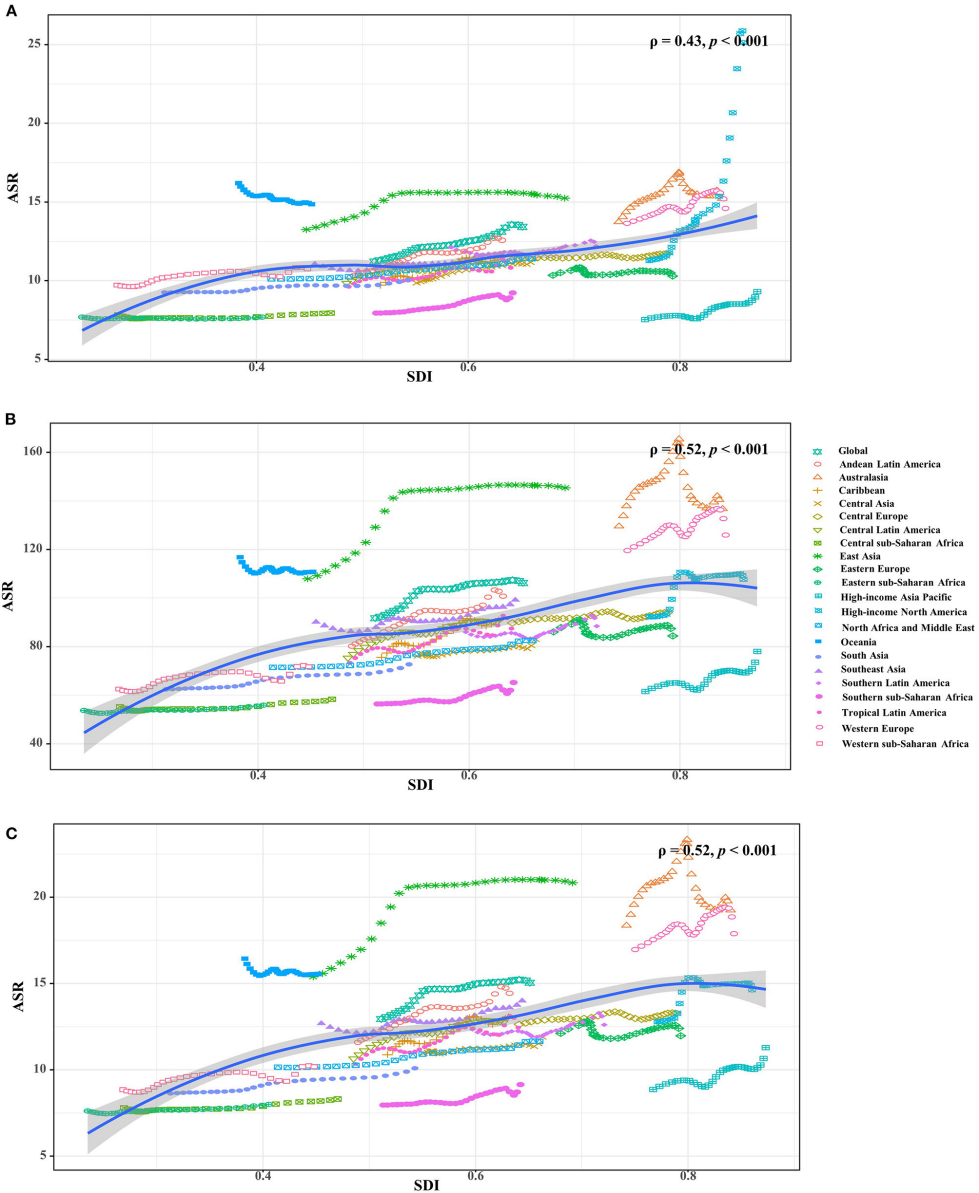
The associations between ASRs of Parkinson's disease and SDI in 2019 among regions. **(A–C)** were that of incidence, prevalence and YLDs, respectively. The points correspond the ASRs of the locations in the years from 1990 to 2019. The association was calculated with Pearson correlation analysis. ASR, age-standardized rate; SDI, socio-demographic index; YLDs, years lived with disability.

Increasing trends in the ASIR of PD occurred in all SDI areas, particularly the high SDI area (EAPC = 1.39, 95% CI: 1.22–1.55). Among 21 geographic regions, East Asia had the largest incident number in 2019 (311.86 × 10^3^), but Oceania had the lowest one (0.87 × 10^3^). The increasing percentage of incidence rose from 27.51% in Eastern Europe to 296.11% in high-income North America. The ASIR varied from Eastern Sub-Saharan Africa (7.70/100,000) to high-income North America (25.14/100,000) in 2019. Eighteen regions presented upward trends in the ASIR, and the largest one occurred in high-income North America (EAPC = 2.74, 95% CI: 2.30–3.18), while downward trend only appeared in Oceania (EAPC = −0.21, 95%CI: −0.25 to −0.18) (Table 1 and Figures 1, 2B,C). A positive association was demonstrated between ASIRs and SDI in 2019 among regions (ρ = 0.43, *p* < 0.001; Figure 3A).

**Figure 4:**
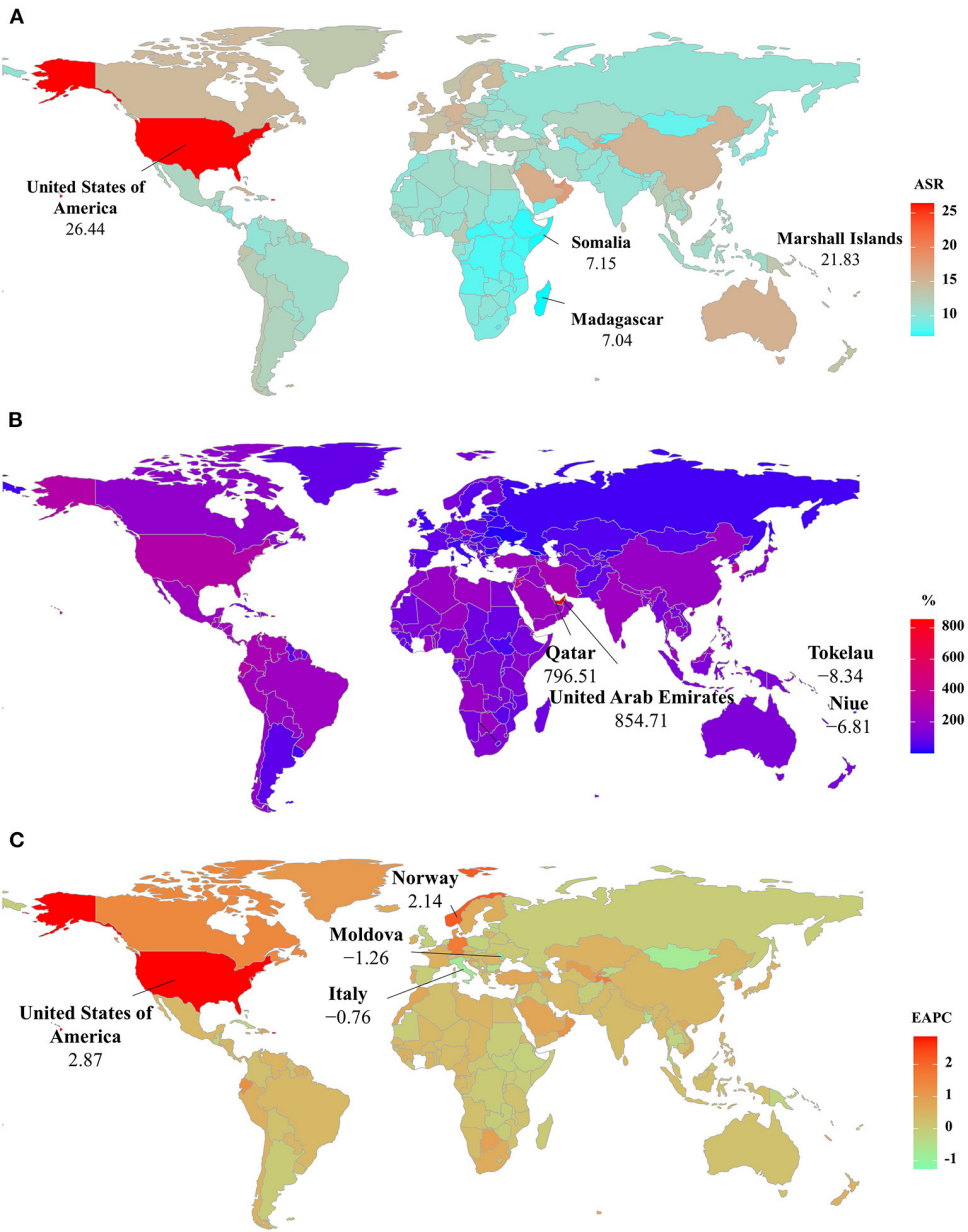
The distribution of ASRs, percentage changes, and EAPCs of Parkinson's disease incidence at the national level. **(A)** was the ASR in 2019; **(B)** was the percentage changes in number between 2000 and 2019; **(C)** was the EAPCs in countries/territories, respectively. Countries/territories with an extreme value were annotated. ASR, age-standardized rate; EAPC, estimated annual percentage change.

The ASIR of PD was heterogeneous among 204 countries/territories, ranging from 7.04/100,000 in Madagascar to 26.44/100,000 in the United States of America in 2019. The largest increasing percentages in incident numbers were seen in Qatar (796.51%) and the United Arab Emirates (854.71%) in 1990–2019, but the decreasing percentages occurred in Tokelau (−8.34%) and Niue (−6.81%). Increasing trends in the ASIR were seen in 150 countries/territories, and the largest ones occurred in the United States of America and Norway, with the respective EAPCs of 2.87 (95% CI: 2.35–3.38) and 2.14 (95% CI: 2.00–2.29). However, decreasing trends were seen in 35 countries, particularly the Republic of Moldova (EAPC = −1.26, 95% CI: −1.33 to −1.19) (Supplementary Table 2 and Figures 4A–C).

### Trends in the PD Prevalence

The global prevalent number of PD increased 155.50% from 1990, and reached 8,511.02 × 10^3^ (95% UI: 7,288.53 × 10^3^-9,841.38 × 10^3^) in 2019. The overall ASR of prevalence was 106.28/100,000 in 2019, and showed an upward trend from 1990 to 2019 (EAPC = 0.52, 95%CI: 0.43–0.61) (Table 1 and Figure 1). Compared with female patients, male patients had a higher prevalence, and showed a larger increasing trend (EAPC = 0.64, 95% CI: 0.53–0.76). Among the age groups, the pronounced high prevalent numbers occurred in patients aged over 65 years (more than 1,000 × 10^3^), and the largest increasing percentage occurred in the age group of over 80 years (203.85%) (Supplementary Table 1 and Supplementary Figure 1A).

All SDI areas presented increasing trends in the ASR of PD prevalence, and the most pronounced one was seen in the middle SDI area (EAPC = 0.75, 95% CI: 0.60–0.90). At the regional level, the largest prevalent number in 2019 appeared in East Asia (2940.41 × 10^3^), while the lowest one occurred in Oceania (6.36 × 10^3^). The increasing percentage of prevalence varied from 27.54% in Eastern Europe to 256.90% in Central Latin America. In 2019, the ASR of prevalence ranged from 55.94/100,000 in Eastern Sub-Saharan Africa to 145.44/100,000 in East Asia. Trends in the ASR of prevalence increased in most regions, particularly East Asia (EAPC = 1.00, 95% CI: 0.74–1.26). Whereas, only Oceania had a minor downward trend (Table 1, Figure 1, and Supplementary Figures 1B,C). A positive association was observed between the ASRs and SDI in 2019 among regions (ρ = 0.52, *p* < 0.001; Figure 3B).

**Figure 5:**
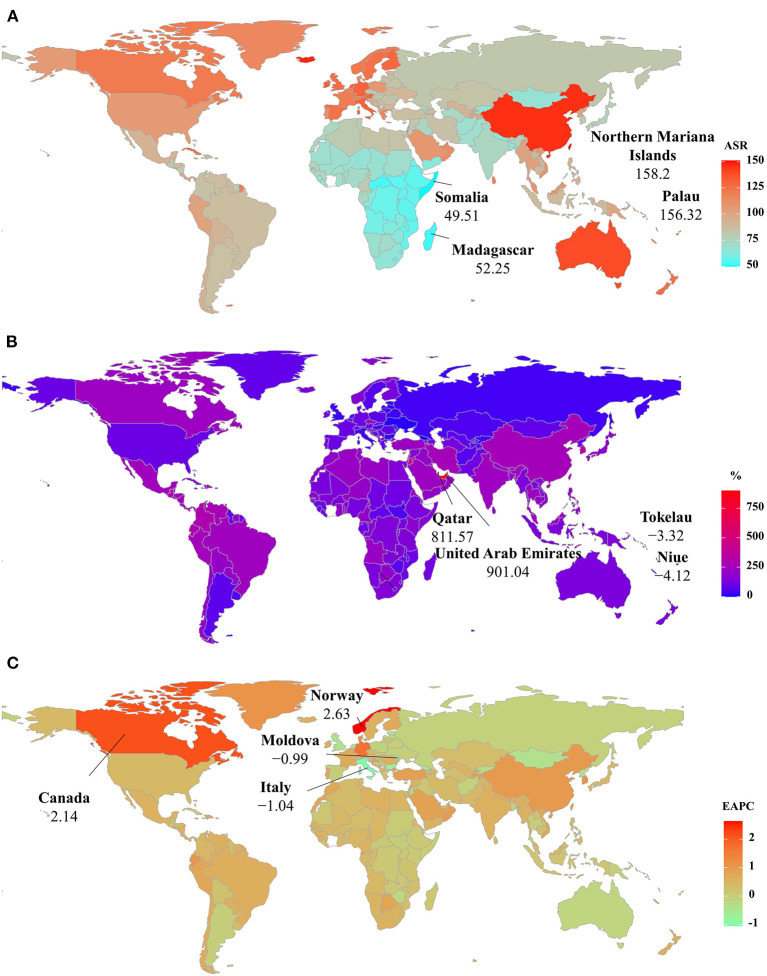
The distribution of ASRs, percentage changes, and EAPCs of Parkinson's disease prevalence at the national level. **(A)** was the ASR in 2019; **(B)** was the percentage changes in number between 2000 and 2019; **(C)** was the EAPCs in countries/territories, respectively. Countries/territories with an extreme value were annotated. ASR, age-standardized rate; EAPC, estimated annual percentage change.

Among 204 countries/territories, the ASR of PD prevalence in 2019 varied from 49.51/100,000 in Somalia to 158.20/100,000 in the Northern Mariana Islands. During 1990–2019, the percentages of the prevalent numbers increased pronouncedly in Qatar and the United Arab Emirates (811.57 and 901.04 9%, respectively), but declined in only four countries, particularly Niue (−4.12%) and Tokelau (−3.32%). Increasing trends in the ASR of prevalence occurred in 164 countries/territories, and the largest one was observed in Norway (EAPC = 2.63, 95% CI: 2.43–2.83), followed by Canada and Germany. However, decreasing trends were observed in 21 countries, particularly Italy and the Republic of Moldova, in which the respective EAPCs were −1.06 (95% CI: −1.35 to −0.77) and −0.99 (95%CI: −1.07 to −0.92) (Supplementary Table 3 and Figures 5A–C).

### Trends in YLDs Caused by PD

In 2019, the number of YLDs caused by PD was 1210.09 × 10^3^ (95% UI: 841.17 × 10^3^-1640.68 × 10^3^) globally, with an increase of 154.73% since 1990. The overall ASR of YLDs reported an increasing trend from 1990 to 2019, with the EAPC of 0.53 (95% CI: 0.44–0.62) (Table 2 and Figure 1). Male patients had a higher burden, and undertook a larger increasing trend than female patients (EAPC = 0.65, 95% CI: 0.54–0.77). Among the age groups, the highest YLD number was seen in the age group of over 80 years (303.81 × 10^3^), and the percentage increased pronouncedly among the patients aged 45 years old (Supplementary Table 1 and Supplementary Figure 2A).

**Table 2:** The changes in YLDs of Parkinson's disease worldwide, and in sexes, SDI areas, and regions, 1990–2019.

	**1990**		**2019**		**1990–2019**	
**Characteristics**	**Number** **×10^**3**^ (95% UI)**	**ASR/100,000** **(95% UI)**	**Number** **×10^**3**^ (95% UI)**	**ASR/100,000** **(95% UI)**	**Percentage change** **(%)**	**EAPC** **(95% CI)**
Overall	475.05 (328.35–648.29)	12.96 (8.96–17.58)	1,210.09 (841.17–1,640.68)	15.06 (10.43–20.35)	154.73	0.53 (0.44–0.62)
**Sex**						
Male	243.33 (166.85–334.32)	15.28 (10.47–20.72)	669.37 (463.92–906.55)	18.40 (12.73–24.80)	175.09	0.65 (0.54–0.77)
Female	231.73 (160.68–317.01)	11.25 (7.79–15.34)	540.73 (377.04–734.62)	12.33 (8.60–16.74)	133.35	0.32 (0.25–0.39)
**SDI**						
Low	16.81 (11.47–23.13)	8.36 (5.70–11.4)	41.89 (28.75–57.64)	9.26 (6.34–12.51)	149.21	0.37 (0.33–0.41)
Low-middle	51.02 (34.64–70.41)	9.85 (6.73–13.38)	146.05 (99.84–201.23)	11.55 (7.96–15.68)	186.27	0.53 (0.49–0.58)
Middle	115.13 (78.14–159.19)	12.78 (8.72–17.47)	374.81 (254.91–520.36)	15.98 (10.99–21.99)	225.54	0.76 (0.60–0.91)
High-middle	146.57 (100.80–200.71)	14.60 (10.08–19.89)	342.67 (235.99–467.85)	16.80 (11.57–22.94)	133.79	0.44 (0.36–0.52)
High	145.26 (101.14–195.71)	13.54 (9.42–18.23)	304.09 (216.62–404.91)	15.25 (10.90–20.28)	109.34	0.51 (0.42–0.59)
**Regions**						
East Asia	119.81 (80.44–167.32)	15.40 (10.46–21.25)	424.83 (286.99–588.17)	20.84 (14.21–28.76)	254.59	1.01 (0.76–1.27)
South Asia	41.80 (28.29–58.01)	8.64 (5.85–11.82)	130.85 (88.79–181.08)	10.10 (6.87–13.85)	213.04	0.52 (0.48–0.56)
Southeast Asia	27.94 (19.24–38.14)	12.68 (8.81–17.18)	76.37 (52.92–104.57)	14.00 (9.76–19.02)	173.35	0.39 (0.32–0.46)
Central Asia	4.65 (3.23–6.40)	10.97 (7.58–14.97)	7.34 (5.04–10.14)	11.90 (8.30–16.24)	57.80	0.19 (0.15–0.23)
High-income Asia Pacific	17.53 (11.91–24.22)	8.88 (6.02–12.13)	53.806 (36.61–73.4)	11.30 (7.68–15.51)	207.26	0.59 (0.48–0.70)
Oceania	0.40 (0.27–0.56)	16.44 (11.25–22.45)	0.91 (0.62–1.28)	15.55 (10.73–21.49)	128.61	−0.07 (−0.12–−0.03)
Australasia	4.35 (3.04–5.89)	18.37 (12.88–24.93)	9.88 (6.71–13.66)	19.26 (13–26.62)	126.93	−0.08 (−0.35–0.18)
Eastern Europe	32.98 (22.52–45.43)	12.12 (8.24–16.60)	42.20 (28.73–58.23)	11.99 (8.18–16.51)	27.97	−0.03 (−0.14–0.07)
Western Europe	103.04 (72.39–138.11)	16.98 (11.97–22.76)	174.35 (122.64–233.60)	17.90 (12.49–24.12)	69.20	0.34 (0.25–0.43)
Central Europe	18.27 (12.67–24.75)	12.64 (8.81–17.12)	30.16 (21.14–40.09)	13.30 (9.35–17.73)	65.08	0.12 (0.08–0.16)
High-income North America	46.91 (31.92–64.04)	12.79 (8.69–17.44)	95.40 (68.61–123.92)	14.65 (10.55–19.08)	103.34	0.54 (0.36–0.73)
Andean Latin America	2.24 (1.52–3.12)	11.60 (7.94–16.05)	7.88 (5.51–10.68)	14.45 (10.1–19.42)	252.09	0.76 (0.69–0.84)
Central Latin America	8.17 (5.63–11.10)	10.65 (7.35–14.38)	29.15 (20.19–39.57)	12.76 (8.86–17.23)	256.90	0.54 (0.46–0.62)
Caribbean	2.75 (1.90–3.72)	10.89 (7.55–14.74)	6.75 (4.72–9.30)	13.10 (9.15–18.01)	145.63	0.60 (0.51–0.69)
Tropical Latin America	8.61 (5.84–11.94)	10.36 (7.03–14.16)	29.34 (20.01–40.30)	12.46 (8.49–17.08)	240.79	0.65 (0.55–0.74)
Southern Latin America	5.67 (3.93–7.8)	12.7 (8.75–17.39)	10.76 (7.48–14.65)	12.62 (8.79–17.18)	89.82	0.18 (0.08–0.28)
Eastern Sub-Saharan Africa	4.96 (3.38–6.84)	7.64 (5.22–10.46)	11.41 (7.72–15.73)	8.01 (5.47–10.78)	129.95	0.19 (0.16–0.22)
Southern Sub-Saharan Africa	1.97 (1.34–2.71)	7.97 (5.45–10.89)	4.59 (3.13–6.21)	9.17 (6.28–12.31)	133.06	0.43 (0.35–0.51)
Western Sub-Saharan Africa	6.57 (4.5–8.96)	8.86 (6.09–11.94)	15.82 (10.97–21.54)	10.19 (7.04–13.62)	140.86	0.50 (0.39–0.60)
North Africa and Middle East	14.97 (10.29–20.74)	10.16 (7.09–13.89)	44.50 (30.65–60.49)	11.66 (8.01–15.7)	197.30	0.54 (0.5–0.58)
Central Sub-Saharan Africa	1.46 (1.00–2.05)	7.81 (5.39–10.84)	3.73 (2.50–5.23)	8.32 (5.68–11.64)	155.11	0.26 (0.20–0.32)

*YLDs, years lived with disability; EAPC, estimated annual percentage change; ASR, age-standardized rate; CI, confidence interval; UI, uncertainty interval; SDI, socio-demographic index*.

The upward trend in the ASR of YLDs occurred in all SDI areas from 1990 to 2019, particularly the middle one (EAPC = 0.76, 95% CI: 0.60–0.91). In terms of geographic regions, the largest number of YLDs was seen in East Asia (424.83 × 10^3^), while the lowest one was in Oceania (0.91 × 10^3^). The percentage of YLDs number increased from 27.97% in Eastern Europe to 256.90% in Central Latin America during 1990–2019. The ASR of YLDs ranged from 8.01/100,000 in Eastern Sub–Saharan Africa to 20.84/100,000 in East Asia. Increasing trends in the ASR of YLDs occurred in most regions, particularly East Asia (EAPC = 1.01, 95% CI: 0.76–1.27). However, a minor decreasing trend was found in Oceania (Table 2, Figure 1, and Supplementary Figures 2B,C). The ASRs of YLDs were positively associated with SDI among the regions in 2019 (ρ = 0.52, *p* < 0.001; Figure 3C).

**Figure 6:**
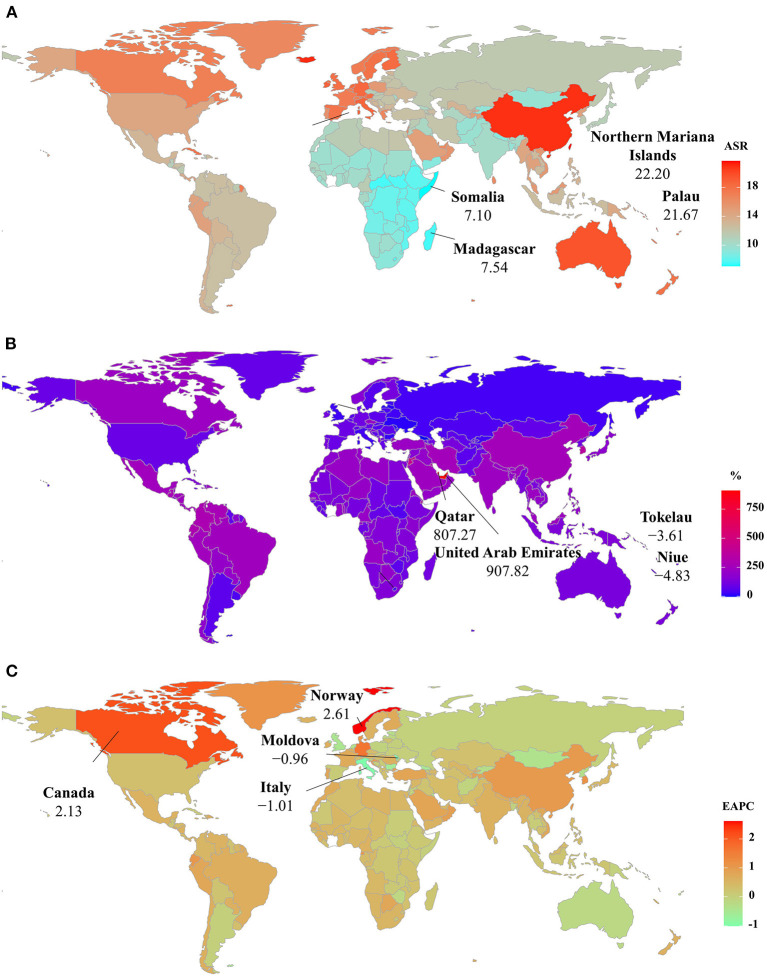
The distribution of ASRs, percentage changes, and EAPCs of YLDs caused by Parkinson's disease at the national level. **(A)** was the ASR in 2019; **(B)** was the percentage changes in number between 2000 and 2019; **(C)** was the EAPCs in countries/territories, respectively. Countries/territories with an extreme value were annotated. YLDs, years lived with disability, ASR, age-standardized rate; EAPC, estimated annual percentage change.

At the national levels, the ASR of YLDs varied across 204 countries/territories, ranging from 7.10/100,000 in Somalia to 22.20/100,000 in the Northern Mariana Islands in 2019. The number of YLDs increased significantly in the United Arab Emirates and Qatar from 1990 to 2019, with the respective percentages of 907.82 and 807.27%. In contrast, decreasing trends occurred in four countries, particularly Niue (−4.83%) and Tokelau (−3.61%). Trends in the ASR of YLDs increased in 123 countries/territories, particularly Norway and Canada, in which the respective EAPCs were 2.61 (95% CI: 2.41–2.80) and 2.13 (95% CI: 1.47–2.79). On the other hand, trends declined in 22 countries/territories, and the most pronounced one occurred in Italy (EAPC = −1.01, 95%CI: −1.30 to −0.72), followed by the Republic of Moldova and Bulgaria (Supplementary Table 4 and Figures 6A–C).

### Analysis of the Influential Factors of EAPC

**Figure 7:**
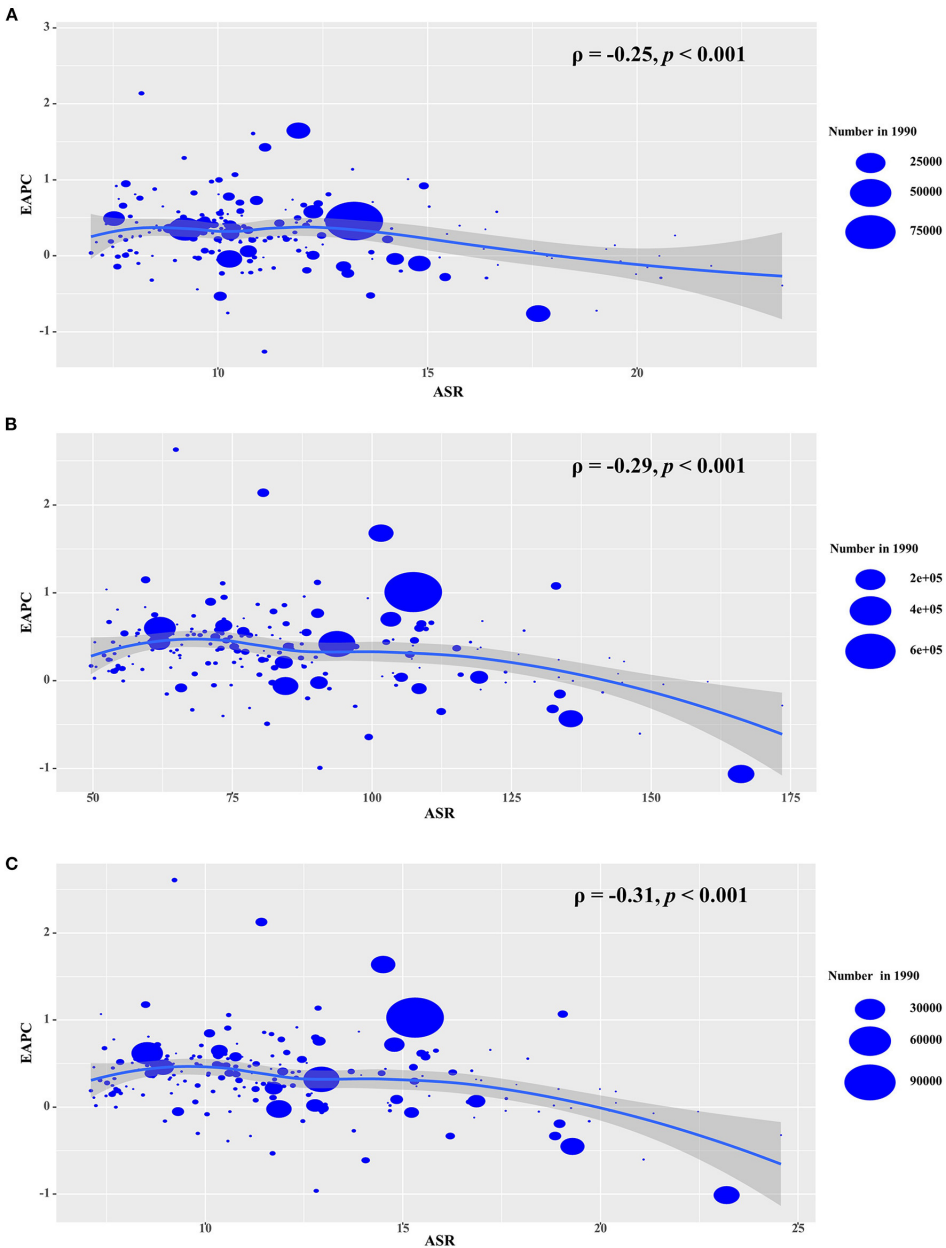
The association s between EAPCs and ASRs in 1990 at the national level. EAPCs of incidence **(A)**, prevalence **(B)**, YLDs **(C)** had negative associations with ASR in 1990, respectively. The association was calculated with Pearson correlation analysis. The size of circle is increased with the numbers in 1990. EAPC, estimated annual percentage change; ASR, age-standardized rate; YLDs, years lived with disability.

The ASR in 1990 served as the disease reservoir at baseline, and the HDI reflected the level of human development, and the availability of health resources in settings, including regions and countries. The EAPCs of the incidence, prevalence, and YLDs had a negative association with the corresponding ASRs in 1990 (ρ = −0.25, *p* < 0.001; ρ = −0.29, *p* < 0.001; ρ = −0.31, *p* < 0.001, respectively; Figures 7A–C). Meanwhile, only the EAPCs of incidence had a positive association with HDI in 2019 (ρ = 0.20, *p* = 0.01; Supplementary Figure 3), which further explained that the trends of the ASIR increased pronouncedly in the high SDI regions and countries from 1990 to 2019.

## Discussion

Different from previous GBD studies, the present study adopted EAPC and ASR to evaluate the changing trends of PD burden over the past 30 years. The results showed that EAPCs and ASRs of the PD burden varied among countries, and most of them showed upward trends from 1990 to 2019.

The rising incident trends of PD were similar to results from previous studies (2, 4, 16), which might be related to population growth and aging (16, 17), genetic predisposition (18), lifestyle changes, and environmental pollution (19–21). Meanwhile, the improved diagnostic methods also led to better identifications of incidence (22, 23). A larger burden and the upward trends of ASIR in males was possibly due to higher occupational factors, unhealthy lifestyles, and so on (24–26). Furthermore, estrogen had potential neuroprotective effects for females (27). The PD incidence was closely related to age, and the pronounced increasing proportions were seen in the groups over 65 years old, supporting the idea that this disease mainly depends on aging trends of ASIR increased pronouncedly in high SDI areas, in where there had a larger aging population (28), decreased total fertility rates (29), and sound health systems (30, 31). The above would also explain the most pronounced upward trends in high SDI countries, including the United States of America, Norway, and Germany. Furthermore, low fertility rates were also a potential reason for the upward trends in these European countries (32). Except for the sheer size of the population, occupational exposure and industrial pollution expedited the increasing trends in low and middle SDI areas (33–36). On the other hand, the decreasing trends of ASIR in Italy might be due to the Mediterranean diet, which has been demonstrated to be related to reduced risk for PD (37–39).

PD is characterized as chronic and non-fatal, and its rising prevalence trend is highly associated with the aging population, which contributed to an increase of about 22% in the ASR of PD prevalence from 1990 to 2016 (16). Because of the advances in treatment and management (40, 41), it was predicted that the PD patients' life would be prolonged by about 3 years, and the ASR of prevalence would increase by 12% over 20 years. Additionally, better estimation methods and studies also potentially promoted the changes in prevalence (42–44), e.g., door-to-door surveys. Among SDI quintiles, middle SDI area had the largest increasing trend in ASR of prevalence, in where there had the largest increase in the number of PD patients (16). Compared to other regions, East Asia presented the largest increasing trends in ASR for prevalence, which was probably driven by population growth and aging (45). Additionally, substantial progress in the health system and social insurance has been achieved (46). The prevalence trends were highly in line with the morbidity of PD, which explained the changing trends among countries. The reason for the decreasing trends of incidence and prevalence in Moldova was due to the rebuilding health and welfare systems (47). Old Moldovans suffered from poverty and lack of health resources, and their low life expectancy was the equivalent of that in the European Union in the 1990's (48).

YLDs reflects the sum of years that an individual lived with disability caused by the non-fatal disease or injury. PD itself is not fatal, and its symptoms generally develop slowly over years. Disability is a critical characteristic of PD, especially in older patients, emphasizing the advantage and necessity of YLDs for the PD study. The rising trend of YLDs was based on the increasing prevalence and prolonged life expectancy of PD patients. Meanwhile, early onset PD with high genetic susceptibility (49, 50), longer disease duration (51), and low adherence to treatment (52, 53), could prolong the course of disability. Some PD is accompanied by stroke (54), dementia (55), and dysphagia (56), which also contributes to the increase in disability. The high prevalence of disability caused by PD was found in low- and middle-income countries, but increasing recognition should strengthen management and access to resources (57). For example, Zhao suggested that it was necessary to carry out genetic testing in early-onset PD patients, particularly those with a family history (58). On the other hand, the minor decreasing trends of YLDs in Oceania were probably explained by a diagnosis of PD at older ages (peaked at 85 years) (59). The pronounced rising trends of YLDs in many high-income countries, including Norway, Canada, and Germany, probably relate to the remarkable increase in disability in later life (60), and complications such as depression, anxiety, and stroke (61, 62). Lower burden of PD was observed in the Mediterranean countries, and decreasing trends in the ASR occurred in Italy and Spain. The decreasing trend of YLDs in Italy benefited from the Mediterranean diet with protective effects on longevity and age-related diseases (63–65). The increasing trends indicated that current strategies were still ill-prepared for the population aging. The findings indicated that active strategies should be adopt to cope with the problems caused by PD, including healthcare and security systems, civil health promotion plan, and healthy lifestyle (66–68).

There were several limitations in this study. First, the quality and quantity of data determined the accuracy and robustness of GBD estimates, which was probably impacted by the potential bias derived from the miscoding and misclassification of disease. Parkinson's disease is accompanied by clinical challenges, including difficulties in diagnosis, misdiagnosis, interference, and so on. Second, the diagnosis and definition of PD had been refined in countries over time, which was the main source of potential bias. Third, the lack of relevant research or data meant we could not fully explain the reason for changing trends in some countries.

In conclusion, the present study provided a comprehensive overview of PD burden and its trends in the incidence, prevalence, and YLDs at the global, regional, and national levels during 1990–2019. Pronounced increasing trends of PD burden ware observed worldwide, and in most regions and countries, indicating that PD is an increasing challenge to global health. The findings highlighted that more effective strategies are needed to decrease the burden of PD, particularly YLDs, when facing with the rapidly growth of aging populations.

## Data Availability Statement

The original contributions presented in the study are included in the article/Supplementary Material, further inquiries can be directed to the corresponding author/s.

## Author Contributions

ZO: project administration and drafting. JP: data analysis and validation. DD and DY: data analysis and visualization. ST and HN: data collection and collation. ZW: supervision and drafting and editing. All authors contributed to the article and approved the submitted version.

## Funding

Guangzhou Health Science and Technology Major Project (Grant No.: 2021A031003).

## Conflict of Interest

The authors declare that the research was conducted in the absence of any commercial or financial relationships that could be construed as a potential conflict of interest.

## Publisher's Note

All claims expressed in this article are solely those of the authors and do not necessarily represent those of their affiliated organizations, or those of the publisher, the editors and the reviewers. Any product that may be evaluated in this article, or claim that may be made by its manufacturer, is not guaranteed or endorsed by the publisher.

## Abbreviations

PD: Parkinson's disease
YLDs: years lived with disability
GBD: Global Burden of Disease
ASR: Age-standardized rate
EAPC: Estimated annual percentage change
UI: Uncertainty interval
CI: Confidence interval
GHDx: Global Health Data Exchange
SDI: Socio-demographic index
HDI: Human Development Index.
