# Supplementary material for: Global Trends in the Incidence, Prevalence, and Years Lived With Disability of Parkinson's Disease in 204 Countries/Territories From 1990 to 2019
Source: Front Public Health. 2021 Dec 7;9:776847. doi: 10.3389/fpubh.2021.776847 (PMC8688697; doi:10.3389/fpubh.2021.776847)
Supplement: Supplementary file 1 [file Data_Sheet_1.docx]

**Supplementary figure 1**. The distribution of Parkinson’s disease prevalence in age groups, SDI areas and geographic regions from 1990 to 2019. (A) was the number in age groups; (B) was the ASR in SDI areas; (C) was the number in geographical regions. ASR, age–standardized rate; SDI, sociodemographic index.

**
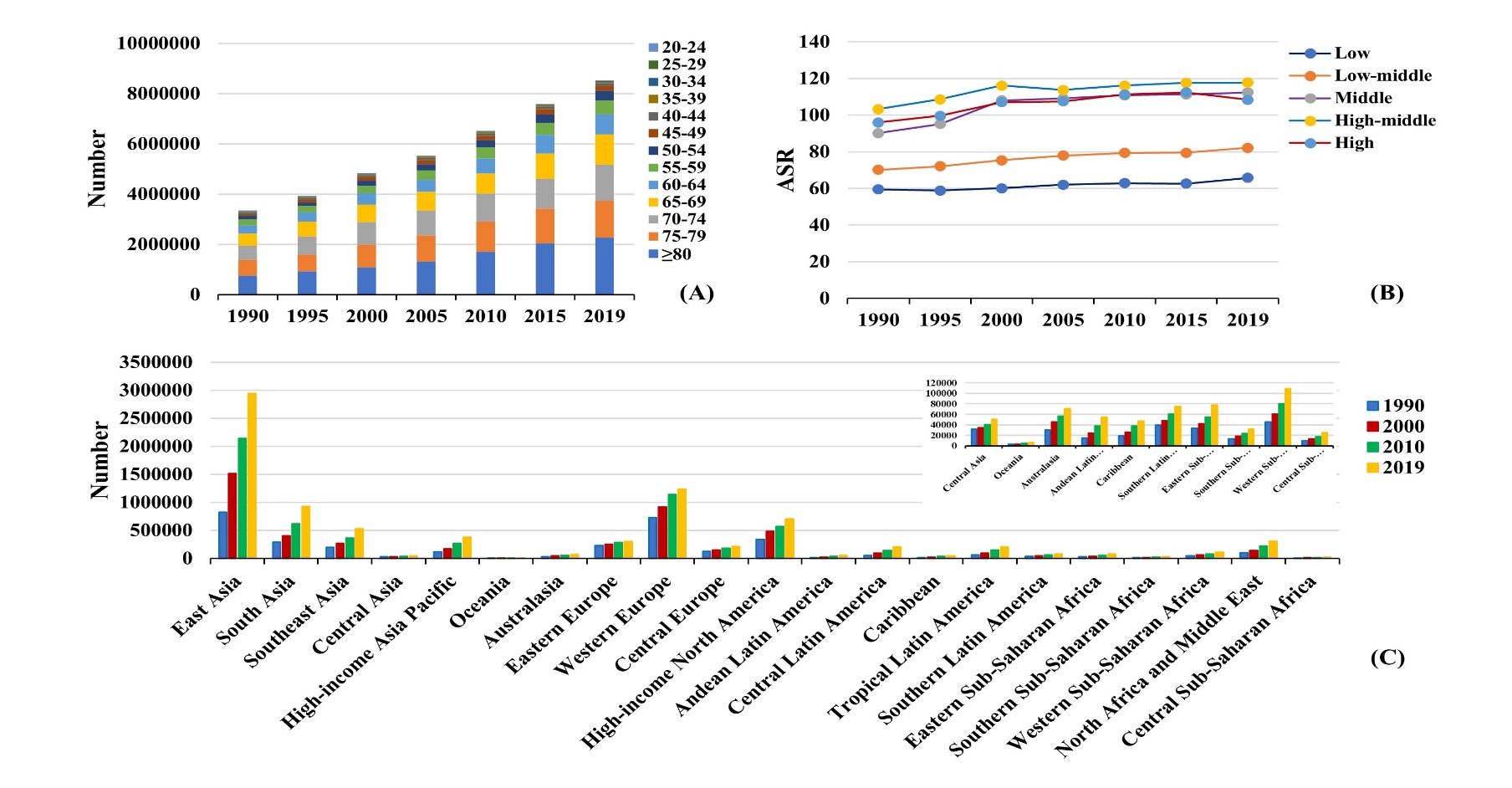
**

**Supplementary figure 2**. The distribution of YLDs caused by Parkinson’s disease in age groups, SDI areas and geographic regions from 1990 to 2019. (A) was the number in age groups; (B) was the ASR in SDI areas; (C) was the number in geographical regions. ASR, age–standardized rate; SDI, sociodemographic index.

**
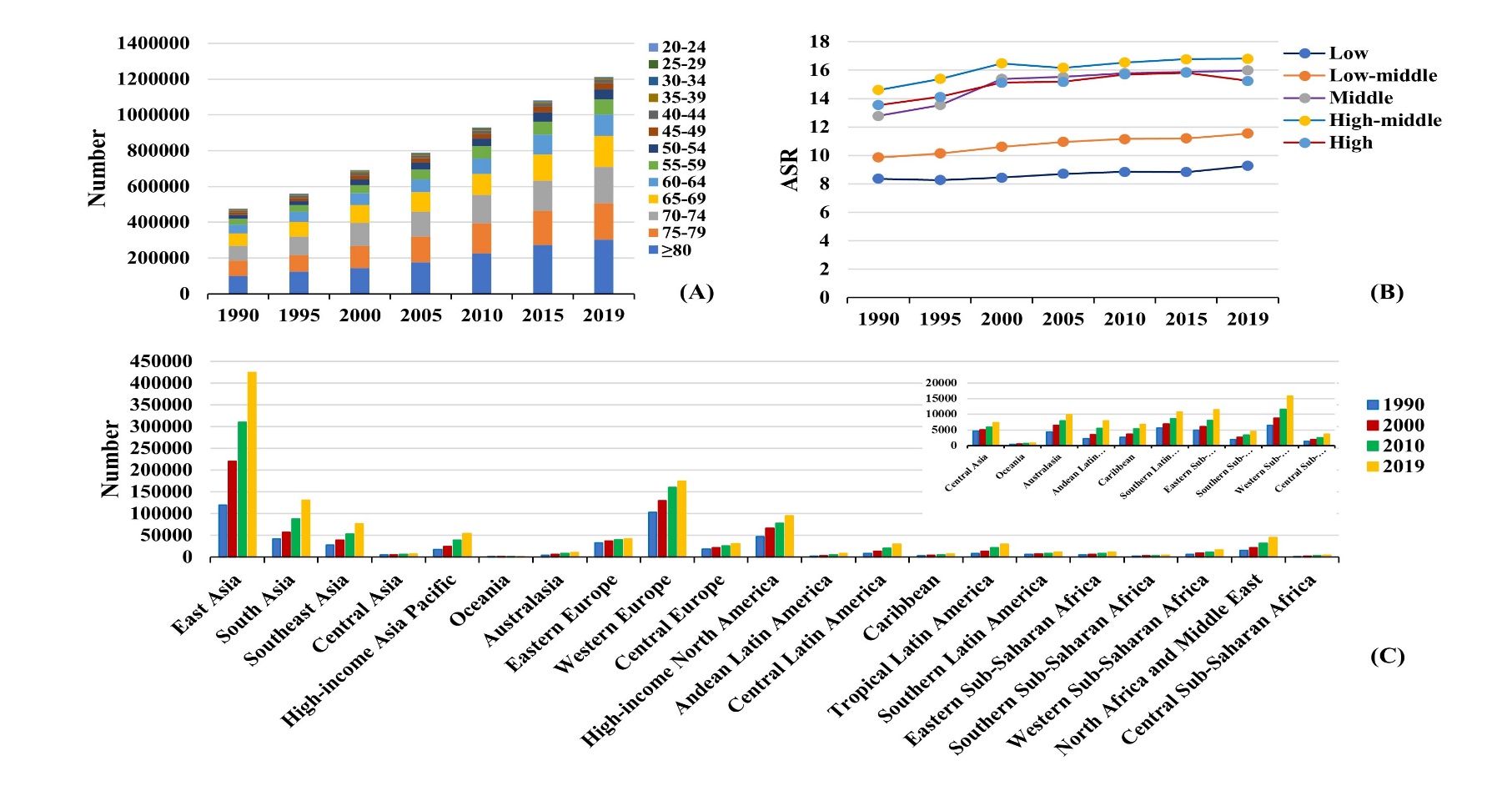
**

**Supplementary figure 3.** The correlation between EAPCs and ASIR in 1990 at the national level. The association was calculated with Pearson correlation analysis. The size of circle is increased with the numbers in 1990. EAPC, estimated annual percentage change; ASIR, age–standardized incidence rate.


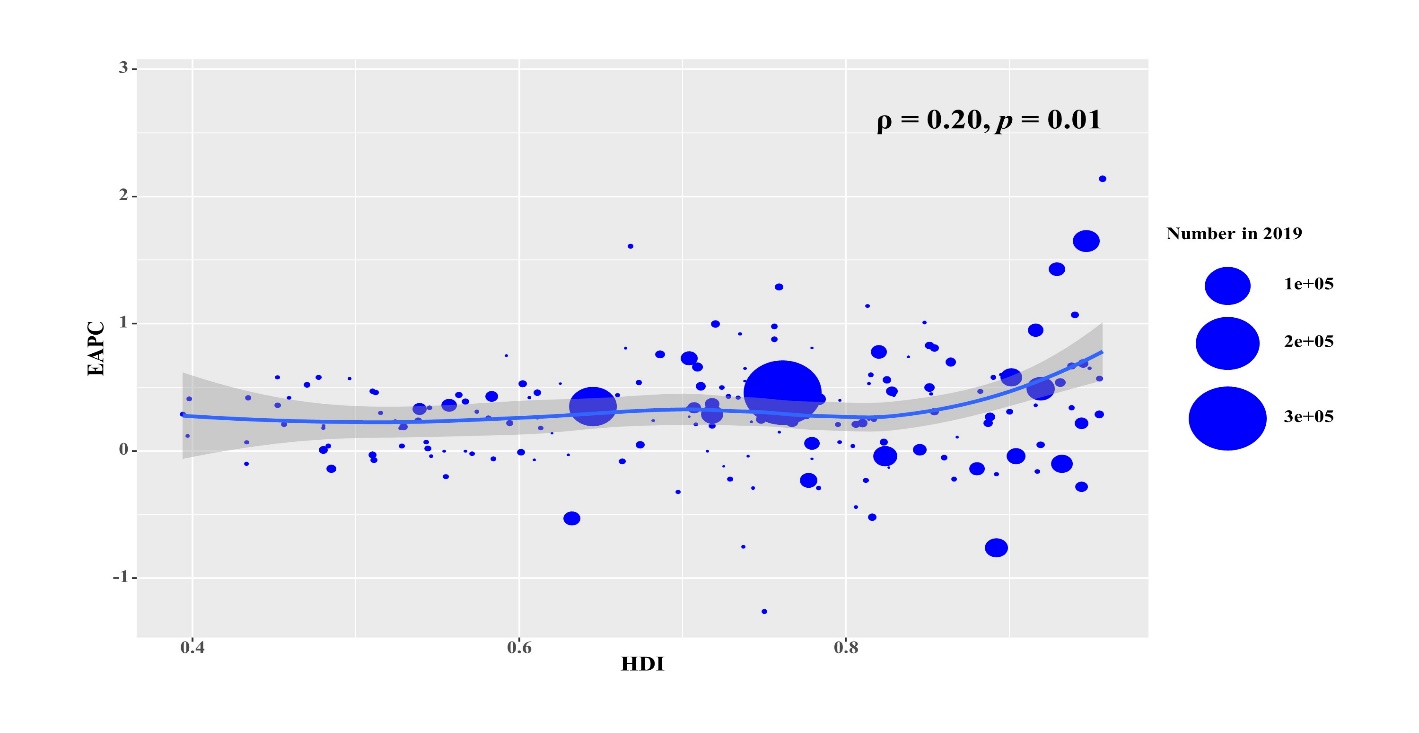


**Supplementary table 1**. The number of Parkinson’s disease in 2019, and the percentage changes in number during the period 1990–2019 in age groups

| **Age Groups** | **Incidence** | | **Prevalence** | | **YLDs** | |
| --- | --- | --- | --- | --- | --- | --- |
|  | Number  ×10^3^(95% UI) | Percentage  change (%) | Number  ×10^3^(95% UI) | Percentage  change (%) | Number  ×10^3^(95% UI) | Percentage  change (%) |
| **20–24** | 0.79(0.28–1.66) | 22.91 | 1.34(0.48–2.83) | 22.91 | 0.22(0.08–0.49) | 22.93 |
| **25–29** | 2.39(0.85–5.05) | 38.35 | 9.3(3.31–19.68) | 38.36 | 1.56(0.52–3.40) | 38.38 |
| **30–34** | 5.10(3.04–7.78) | 64.89 | 26.97(12.99–51.02) | 60.73 | 4.53(1.99–8.95) | 60.75 |
| **35–39** | 7.95(3.33–13.6) | 63.26 | 54.89(32.71–83.9) | 59.94 | 9.22(4.89–15.55) | 59.95 |
| **40–44** | 15.73(10.46–21.8) | 101.87 | 102.2(65.2–148.66) | 88.46 | 17.04(9.53–28.09) | 87.49 |
| **45–49** | 29.93(17.6–43.69) | 166.76 | 209.86(150.76–279.08) | 147.48 | 33.66(20.69–51.89) | 144.77 |
| **50–54** | 49.98(36.49–66.25) | 169.24 | 374.87(276.3–493.91) | 167.05 | 58.34(36.45–88.05) | 165.49 |
| **55–59** | 66.39(42.68–98.3) | 148.52 | 552.91(429.92–710.54) | 151.69 | 83.34(54.24–122.99) | 152.82 |
| **60–64** | 109.65(83.16–139.01) | 128.6 | 802.18(624.84–1041.49) | 137.79 | 118.65(78.12–173.92) | 139.27 |
| **65–69** | 163.02(116.4–222.35) | 138.91 | 1205.78(957.53–1492.4) | 146.49 | 174.57(114.68–248.76) | 147.37 |
| **70–74** | 191.4(151.69–231.77) | 162.45 | 1434.18(1149.67–1776.09) | 153.26 | 203.08(134.04–282.99) | 154.39 |
| **75–79** | 192.39(145.03–246.78) | 153.35 | 1460.27(1194.1–1735.63) | 131.35 | 202.07(140.62–274) | 131.69 |
| **≥ 80** | 247.00(201.03–293.59) | 221.67 | 2276.28(1870.16–2715.47) | 203.85 | 303.81(212.22–406.65) | 203.27 |

YLDs, years lived with disability

**Supplementary table 2**. The percentage changes and EAPCs of Parkinson’s disease incidence at the national level from 1990 to 2019.

|  | **1990** | | **2019** | | **1990-2019** | |
| --- | --- | --- | --- | --- | --- | --- |
| **Characteristics** | Number  ×10^3^ (95% UI) | ASR/100,000  (95% UI) | Number  ×10^3^ (95% UI) | ASR/100,000  (95% UI) | Percentage change (%) | EAPC  (95%CI) |
| Afghanistan | 0.68(0.59–0.78) | 10.76(9.61–12.06) | 1.1(0.98–1.24) | 10.46(9.49–11.57) | 63.19 | −0.07(−0.10–−0.04) |
| Albania | 0.22(0.2–0.25) | 11.85(10.59–13.24) | 0.56(0.49–0.63) | 12.58(11.14–14.01) | 148.68 | 0.21(0.18–0.24) |
| Algeria | 1.07(0.93–1.22) | 10.28(9.2–11.52) | 3.21(2.84–3.68) | 10.96(9.75–12.34) | 200.83 | 0.25(0.22–0.29) |
| American Samoa | 0(0–0) | 20.24(18.2–22.67) | 0.01(0.01–0.01) | 18.84(16.94–20.92) | 113.73 | −0.15(−0.19–−0.1) |
| Andorra | 0.01(0.01–0.01) | 12.1(10.7–13.94) | 0.02(0.02–0.02) | 12.5(10.8–14.37) | 169.32 | 0.11(0.08–0.15) |
| Angola | 0.24(0.2–0.27) | 7.66(6.83–8.63) | 0.72(0.62–0.83) | 8.11(7.16–9.2) | 206.01 | 0.26(0.24–0.29) |
| Antigua and Barbuda | 0.01(0.01–0.01) | 11.61(10.46–12.86) | 0.01(0.01–0.01) | 14.09(12.37–15.76) | 100.4 | 0.61(0.51–0.72) |
| Argentina | 3.91(3.52–4.4) | 12.26(11.11–13.6) | 6.64(6.00–7.42) | 11.96(10.81–13.37) | 70.01 | 0.01(−0.07–0.09) |
| Armenia | 0.23(0.2–0.25) | 9.39(8.51–10.35) | 0.41(0.36–0.46) | 10.05(8.79–11.2) | 82.6 | 0.27(0.21–0.33) |
| Australia | 2.77(2.49–3.11) | 14.04(12.62–15.66) | 6.60(5.75–7.62) | 15.5(13.39–17.94) | 138.26 | 0.22(0.02–0.43) |
| Austria | 1.57(1.42–1.74) | 12.63(11.52–14.09) | 2.77(2.46–3.09) | 14.87(13.07–16.68) | 76.03 | 0.47(0.41–0.54) |
| Azerbaijan | 0.43(0.39–0.49) | 9.84(8.74–10.92) | 0.94(0.82–1.06) | 13.16(11.9–14.44) | 115.34 | 0.98(0.95–1.01) |
| Bahamas | 0.02(0.01–0.02) | 11.44(10.36–12.69) | 0.04(0.04–0.05) | 12.62(11.41–14.16) | 171.94 | 0.25(0.17–0.32) |
| Bahrain | 0.02(0.01–0.02) | 12.13(10.83–13.5) | 0.09(0.08–0.11) | 13.67(12.01–15.51) | 466.83 | 0.45(0.41–0.49) |
| Bangladesh | 4.09(3.62–4.63) | 10.05(9.02–11.23) | 10.86(9.58–12.35) | 8.92(7.98–10.11) | 165.61 | −0.53(−0.57–−0.49) |
| Barbados | 0.03(0.03–0.04) | 10.56(9.64–11.71) | 0.06(0.06–0.07) | 12.77(11.58–14.06) | 88.29 | 0.53(0.42–0.63) |
| Belarus | 1.24(1.09–1.42) | 9.68(8.52–11.04) | 1.63(1.46–1.87) | 10.13(9.1–11.61) | 31.1 | 0.07(0.01–0.12) |
| Belgium | 2.06(1.83–2.34) | 12.85(11.45–14.46) | 3.3(2.89–3.62) | 14.04(12.16–15.71) | 60.02 | 0.54(0.47–0.6) |
| Belize | 0.01(0.01–0.01) | 10.36(9.15–11.67) | 0.03(0.03–0.03) | 11.77(10.46–13.12) | 212.65 | 0.35(0.22–0.48) |
| Benin | 0.18(0.16–0.2) | 10.19(9.14–11.23) | 0.45(0.4–0.5) | 11.17(9.97–12.43) | 147.41 | 0.34(0.26–0.41) |
| Bermuda | 0.01(0.01–0.01) | 15.28(13.77–17.06) | 0.02(0.02–0.02) | 15.46(14.19–17.85) | 131.08 | −0.02(−0.11–0.08) |
| Bhutan | 0.02(0.01–0.02) | 8.53(7.56–9.53) | 0.05(0.04–0.06) | 9.67(8.64–10.78) | 192.12 | 0.4(0.38–0.43) |
| Bolivia | 0.32(0.29–0.36) | 11.11(10.04–12.18) | 0.97(0.87–1.09) | 11.95(10.77–13.26) | 204.22 | 0.2(0.16–0.24) |
| Bosnia and Herzegovina | 0.45(0.4–0.5) | 11.92(10.75–13.2) | 0.74(0.66–0.84) | 12.07(10.82–13.59) | 66.31 | 0.07(0.04–0.09) |
| Botswana | 0.04(0.03–0.04) | 7.57(6.75–8.54) | 0.11(0.09–0.12) | 9.89(8.78–11) | 201.49 | 0.92(0.86–0.98) |
| Brazil | 7.68(6.41–8.97) | 9.65(8.07–11.2) | 24.9(20.95–28.99) | 10.84(9.09–12.62) | 224.05 | 0.42(0.34–0.49) |
| Brunei Darussalam | 0.01(0.01–0.01) | 11.77(10.47–13.27) | 0.03(0.03–0.04) | 14.07(12.47–15.68) | 257.93 | 0.74(0.64–0.84) |
| Bulgaria | 1.66(1.49–1.84) | 13.64(12.48–14.96) | 1.95(1.76–2.21) | 12.49(11.31–14.05) | 17.23 | −0.52(−0.6–−0.44) |
| Burkina Faso | 0.34(0.3–0.39) | 9.68(8.67–10.83) | 0.77(0.68–0.88) | 10.58(9.48–11.82) | 126.33 | 0.36(0.29–0.44) |
| Burundi | 0.17(0.15–0.19) | 7.91(7.01–8.85) | 0.29(0.26–0.34) | 7.99(7.11–9) | 78.15 | 0.07(0.03–0.1) |
| Cabo Verde | 0.02(0.02–0.02) | 8.02(7–9.23) | 0.04(0.03–0.04) | 9.81(8.68–10.88) | 101.05 | 0.81(0.75–0.88) |
| Cambodia | 0.4(0.35–0.46) | 10.31(9.07–11.69) | 1.15(1.01–1.29) | 10.68(9.54–11.99) | 185.18 | 0.22(0.13–0.31) |
| Cameroon | 0.4(0.35–0.44) | 11.45(10.38–12.61) | 1.19(1.06–1.33) | 12.82(11.53–14.08) | 200.97 | 0.44(0.37–0.52) |
| Canada | 3.67(3.34–4.08) | 11.12(10.12–12.31) | 10.42(9.2–11.87) | 14.73(12.96–16.87) | 183.53 | 1.43(1.01–1.85) |
| Central African Republic | 0.07(0.06–0.08) | 7.48(6.6–8.41) | 0.13(0.11–0.15) | 7.57(6.74–8.54) | 84.17 | 0.12(0.1–0.15) |
| Chad | 0.24(0.21–0.27) | 9.52(8.53–10.65) | 0.48(0.42–0.54) | 10.55(9.5–11.76) | 100.27 | 0.41(0.35–0.47) |
| Chile | 1.11(1.02–1.24) | 11.89(10.93–13.11) | 3.11(2.73–3.53) | 12.9(11.37–14.64) | 179.18 | 0.5(0.43–0.57) |
| China | 99.23(82.52–116.98) | 13.24(11.16–15.46) | 301.53(250.66–352.35) | 15.27(12.82–17.78) | 203.85 | 0.46(0.3–0.62) |
| Colombia | 1.49(1.34–1.64) | 9.41(8.53–10.38) | 5.38(4.86–6.05) | 10.11(9.13–11.4) | 262.22 | 0.23(0.16–0.31) |
| Comoros | 0.02(0.01–0.02) | 8.43(7.51–9.4) | 0.04(0.03–0.04) | 8.41(7.48–9.51) | 121.83 | 0(−0.01–0.02) |
| Congo | 0.07(0.06–0.08) | 8.35(7.45–9.27) | 0.19(0.17–0.22) | 8.94(8.01–9.96) | 158.13 | 0.31(0.28–0.35) |
| Cook Islands | 0(0–0) | 19.97(17.7–22.51) | 0(0–0.01) | 17.99(15.8–20.41) | 95.18 | −0.24(−0.28–−0.2) |
| Costa Rica | 0.18(0.16–0.2) | 10.77(9.59–11.92) | 0.59(0.52–0.66) | 11.69(10.25–13.15) | 227.5 | 0.23(0.2–0.27) |
| Croatia | 0.71(0.62–0.8) | 11.23(9.97–12.63) | 1.14(1.01–1.27) | 12.25(10.86–13.65) | 61.26 | 0.24(0.2–0.28) |
| Cuba | 0.97(0.88–1.08) | 9.42(8.55–10.47) | 2.27(2.01–2.55) | 11.69(10.38–13.08) | 134.18 | 0.83(0.73–0.93) |
| Cyprus | 0.13(0.11–0.15) | 16.4(14.28–18.71) | 0.3(0.26–0.34) | 15.15(13.22–17.23) | 129.05 | −0.29(−0.32–−0.25) |
| Czechia | 1.42(1.28–1.61) | 9.99(9.01–11.34) | 2.41(2.18–2.62) | 10.83(9.73–11.84) | 70.18 | 0.22(0.17–0.28) |
| Côte d'Ivoire | 0.33(0.29–0.37) | 11.83(10.73–12.95) | 1(0.88–1.13) | 12.7(11.49–14.06) | 203.12 | 0.31(0.24–0.37) |
| Democratic People's Republic of Korea | 1.75(1.48–2.03) | 12.12(10.5–13.81) | 3.55(3.08–4.04) | 11.4(9.97–12.9) | 103.16 | −0.19(−0.26–−0.13) |
| Democratic Republic of the Congo | 0.97(0.84–1.12) | 7.79(6.92–8.82) | 2.31(1.98–2.65) | 7.77(6.84–8.84) | 137.39 | 0.01(−0.05–0.08) |
| Denmark | 0.88(0.79–0.98) | 10.4(9.3–11.54) | 1.58(1.41–1.72) | 13.24(11.73–14.6) | 79.46 | 1.07(1–1.13) |
| Djibouti | 0.01(0.01–0.01) | 7.95(7.02–9.07) | 0.04(0.03–0.05) | 8.46(7.43–9.67) | 367.65 | 0.24(0.22–0.26) |
| Dominica | 0.01(0.01–0.01) | 11.73(10.6–13.14) | 0.01(0.01–0.01) | 12.84(11.49–14.48) | 30.72 | 0.23(0.12–0.35) |
| Dominican Republic | 0.29(0.26–0.33) | 8.48(7.62–9.47) | 0.99(0.89–1.08) | 11.13(10.01–12.22) | 240.05 | 0.86(0.79–0.93) |
| Ecuador | 0.46(0.41–0.51) | 9.18(8.25–10.16) | 1.9(1.69–2.12) | 13.32(11.89–14.84) | 315.22 | 1.29(1.22–1.36) |
| Egypt | 2.59(2.25–2.94) | 10.72(9.52–12) | 6.28(5.47–7.21) | 12.02(10.53–13.56) | 142.41 | 0.34(0.31–0.36) |
| El Salvador | 0.27(0.24–0.3) | 9.61(8.6–10.54) | 0.7(0.63–0.77) | 11.44(10.3–12.76) | 158.74 | 0.54(0.48–0.6) |
| Equatorial Guinea | 0.01(0.01–0.01) | 7.62(6.82–8.52) | 0.04(0.03–0.04) | 9.08(8.02–10.25) | 197.8 | 0.75(0.7–0.79) |
| Eritrea | 0.06(0.05–0.06) | 7.33(6.46–8.31) | 0.18(0.15–0.2) | 8.31(7.36–9.34) | 219.82 | 0.42(0.4–0.44) |
| Estonia | 0.23(0.2–0.26) | 11(9.76–12.62) | 0.29(0.26–0.33) | 10.68(9.39–12.29) | 28.32 | −0.18(−0.23–−0.12) |
| Eswatini | 0.02(0.02–0.02) | 7.96(7.07–8.85) | 0.04(0.04–0.05) | 8.77(7.84–9.8) | 120.5 | 0.26(0.22–0.31) |
| Ethiopia | 1.25(1.04–1.47) | 7.59(6.37–8.86) | 2.7(2.27–3.14) | 7.35(6.16–8.58) | 115.99 | −0.14(−0.17–−0.12) |
| Fiji | 0.06(0.05–0.07) | 20.57(18.28–23.09) | 0.12(0.1–0.13) | 18.39(16.39–20.51) | 98.03 | −0.29(−0.32–−0.26) |
| Finland | 0.88(0.76–0.98) | 12.04(10.44–13.47) | 1.86(1.63–2.12) | 14.69(12.85–16.96) | 111.95 | 0.67(0.64–0.7) |
| France | 10.63(9.66–11.72) | 12.27(11.05–13.57) | 19.68(17.92–21.85) | 13.95(12.64–15.66) | 85.04 | 0.58(0.49–0.67) |
| Gabon | 0.05(0.04–0.05) | 9.61(8.58–10.73) | 0.09(0.08–0.11) | 10.47(9.46–11.78) | 96.14 | 0.34(0.31–0.36) |
| Gambia | 0.03(0.03–0.03) | 9.97(8.92–11.17) | 0.1(0.09–0.11) | 11.65(10.51–12.94) | 244.3 | 0.57(0.51–0.63) |
| Georgia | 0.6(0.52–0.69) | 10.09(8.89–11.41) | 0.6(0.54–0.66) | 9.78(8.84–10.7) | 0.34 | −0.23(−0.27–−0.19) |
| Germany | 15.52(13.95–17.31) | 11.92(10.66–13.3) | 30.52(26.19–34.96) | 15.31(13.11–17.4) | 96.62 | 1.65(1.32–1.97) |
| Ghana | 0.44(0.39–0.5) | 9.03(8.05–10.19) | 1.33(1.17–1.48) | 10.17(9.13–11.25) | 202.57 | 0.46(0.36–0.56) |
| Greece | 1.94(1.73–2.18) | 12.47(11.13–13.88) | 3.31(2.93–3.72) | 13.37(11.86–15.2) | 70.2 | 0.27(0.22–0.32) |
| Greenland | 0(0–0) | 16.33(14.49–18.4) | 0.01(0.01–0.01) | 16.9(15.12–18.94) | 135.84 | 0.09(0.06–0.13) |
| Grenada | 0.01(0.01–0.01) | 10.01(9.14–11.09) | 0.01(0.01–0.02) | 13.1(11.92–15.15) | 75.63 | 0.81(0.75–0.88) |
| Guam | 0.01(0.01–0.01) | 19.03(16.67–21.34) | 0.03(0.02–0.03) | 15.29(13.5–17.24) | 134.09 | −0.72(−0.77–−0.67) |
| Guatemala | 0.3(0.26–0.34) | 10.12(9.07–11.27) | 1.07(0.96–1.19) | 10.18(9.15–11.26) | 253.53 | −0.08(−0.16–0) |
| Guinea | 0.3(0.27–0.34) | 10.49(9.49–11.71) | 0.57(0.51–0.64) | 12.19(11.11–13.42) | 90.17 | 0.58(0.5–0.65) |
| Guinea–Bissau | 0.03(0.03–0.04) | 10.53(9.44–11.8) | 0.06(0.05–0.07) | 11.02(10.03–12.13) | 79.32 | 0.2(0.13–0.26) |
| Guyana | 0.04(0.03–0.04) | 10.92(9.86–12.26) | 0.07(0.06–0.07) | 11.93(10.93–13.19) | 77.02 | 0.24(0.15–0.32) |
| Haiti | 0.27(0.24–0.31) | 9.9(8.85–11.08) | 0.68(0.6–0.77) | 11.52(10.35–12.92) | 150.97 | 0.47(0.4–0.55) |
| Honduras | 0.19(0.17–0.21) | 10.07(9.12–11.12) | 0.63(0.56–0.71) | 11.33(10.03–12.68) | 235.27 | 0.36(0.32–0.41) |
| Hungary | 1.46(1.33–1.62) | 9.64(8.82–10.64) | 2.12(1.9–2.34) | 10.42(9.34–11.65) | 44.6 | 0.31(0.25–0.36) |
| Iceland | 0.04(0.04–0.05) | 15.04(13.35–16.74) | 0.1(0.09–0.12) | 17.74(15.72–20.64) | 126.55 | 0.65(0.61–0.69) |
| India | 33(27.55–38.86) | 9.22(7.7–10.75) | 106.9(89.26–125.59) | 10.26(8.54–11.97) | 223.9 | 0.35(0.31–0.38) |
| Indonesia | 8.59(7.23–10.01) | 10.29(8.67–12.04) | 20.78(17.34–24.17) | 11.02(9.25–12.88) | 141.79 | 0.29(0.23–0.34) |
| Iran  (Islamic Republic of) | 2.09(1.75–2.43) | 9.89(8.29–11.53) | 7.44(6.28–8.66) | 11.24(9.41–13.21) | 256.69 | 0.41(0.36–0.47) |
| Iraq | 0.69(0.61–0.78) | 9.84(8.7–11.09) | 1.95(1.71–2.21) | 10(8.83–11.26) | 183.59 | 0.05(0.03–0.08) |
| Ireland | 0.55(0.49–0.62) | 13.02(11.6–14.43) | 1.16(1.01–1.33) | 15.15(13.29–17.41) | 108.97 | 0.57(0.53–0.61) |
| Israel | 0.68(0.61–0.77) | 13.65(12.28–15.36) | 1.6(1.43–1.8) | 13.57(12.13–15.32) | 134.79 | 0.05(0–0.1) |
| Italy | 16.43(13.48–19.45) | 17.64(14.65–20.66) | 22.41(18.34–26.68) | 14.93(12.45–17.64) | 36.4 | −0.76(−0.98–−0.55) |
| Jamaica | 0.18(0.16–0.2) | 9.68(8.66–10.93) | 0.33(0.3–0.36) | 11.03(10.09–12.19) | 85.06 | 0.42(0.29–0.55) |
| Japan | 12.64(10.5–14.92) | 7.51(6.29–8.82) | 34.32(28.1–40.85) | 9.11(7.62–10.7) | 171.66 | 0.49(0.39–0.59) |
| Jordan | 0.11(0.1–0.12) | 10.57(9.46–11.7) | 0.56(0.49–0.64) | 10.25(9.06–11.64) | 408.03 | −0.22(−0.29–−0.16) |
| Kazakhstan | 1.16(1.03–1.31) | 10.05(8.96–11.25) | 1.78(1.59–2.05) | 11.71(10.68–13.19) | 53.54 | 0.56(0.48–0.65) |
| Kenya | 0.54(0.46–0.64) | 7.6(6.38–8.88) | 1.42(1.2–1.65) | 7.61(6.39–8.89) | 161.56 | −0.01(−0.04–0.01) |
| Kiribati | 0.01(0–0.01) | 17.96(16.13–20.08) | 0.01(0.01–0.01) | 17.37(15.7–19.08) | 70.79 | −0.03(−0.06–0) |
| Kuwait | 0.05(0.04–0.06) | 9.5(8.27–10.9) | 0.19(0.17–0.22) | 8.41(7.38–9.63) | 288.15 | −0.44(−0.49–−0.39) |
| Kyrgyzstan | 0.24(0.22–0.27) | 8.41(7.42–9.46) | 0.32(0.29–0.36) | 7.9(7.1–8.76) | 32.22 | −0.32(−0.35–−0.3) |
| Lao People's Democratic Republic | 0.19(0.17–0.22) | 10.53(9.45–11.84) | 0.42(0.37–0.48) | 11.06(9.83–12.48) | 120.73 | 0.18(0.11–0.24) |
| Latvia | 0.39(0.35–0.44) | 10.78(9.79–12) | 0.43(0.39–0.47) | 10.28(9.23–11.52) | 9.75 | −0.22(−0.27–−0.16) |
| Lebanon | 0.18(0.16–0.21) | 9.38(8.31–10.56) | 0.54(0.48–0.6) | 10.2(9.18–11.42) | 189.94 | 0.36(0.33–0.4) |
| Lesotho | 0.06(0.05–0.07) | 7.09(6.36–7.91) | 0.08(0.07–0.09) | 7.69(6.89–8.49) | 34.08 | 0.18(0.13–0.24) |
| Liberia | 0.1(0.09–0.11) | 10.04(9.03–11.24) | 0.17(0.15–0.19) | 10.53(9.39–11.8) | 76.94 | 0.18(0.1–0.27) |
| Libya | 0.17(0.15–0.19) | 10.07(9.02–11.21) | 0.51(0.46–0.57) | 11.25(9.95–12.53) | 208.55 | 0.5(0.45–0.55) |
| Lithuania | 0.41(0.36–0.47) | 9.06(8.01–10.32) | 0.61(0.56–0.68) | 10.21(9.29–11.43) | 49.11 | 0.47(0.43–0.51) |
| Luxembourg | 0.08(0.07–0.09) | 14.14(12.76–15.85) | 0.16(0.14–0.18) | 15.91(14.11–17.94) | 101.01 | 0.36(0.27–0.45) |
| Madagascar | 0.31(0.27–0.35) | 6.96(6.16–7.9) | 0.62(0.53–0.72) | 7.04(6.2–7.96) | 100.82 | 0.04(0.01–0.06) |
| Malawi | 0.26(0.22–0.3) | 8.04(7.18–9.05) | 0.51(0.45–0.57) | 8.1(7.19–9.13) | 97.02 | 0.04(0.02–0.06) |
| Malaysia | 0.96(0.82–1.11) | 11.62(10.05–13.39) | 3.02(2.53–3.53) | 12.1(10.38–13.92) | 215.55 | 0.22(0.17–0.28) |
| Maldives | 0.01(0.01–0.01) | 13.68(12.07–15.25) | 0.04(0.03–0.04) | 13.08(11.45–14.71) | 274.05 | −0.04(−0.13–0.05) |
| Mali | 0.31(0.27–0.35) | 9.36(8.38–10.5) | 0.73(0.65–0.81) | 10.48(9.46–11.57) | 138.29 | 0.42(0.33–0.51) |
| Malta | 0.06(0.05–0.07) | 13.34(11.88–15.08) | 0.15(0.13–0.17) | 15.57(13.86–17.66) | 164.67 | 0.6(0.56–0.65) |
| Marshall Islands | 0(0–0) | 20.9(18.8–23.45) | 0.01(0–0.01) | 21.83(19.71–24.11) | 92.68 | 0.27(0.23–0.31) |
| Mauritania | 0.09(0.08–0.1) | 10.28(9.42–11.41) | 0.19(0.17–0.21) | 10.32(9.3–11.56) | 109.33 | −0.04(−0.13–0.05) |
| Mauritius | 0.08(0.07–0.09) | 12.12(10.67–13.94) | 0.21(0.18–0.24) | 12.28(10.59–14.18) | 153.96 | 0.04(−0.01–0.09) |
| Mexico | 3.95(3.32–4.61) | 10.29(8.62–11.98) | 13.07(11.01–15.22) | 11.75(9.89–13.7) | 230.56 | 0.41(0.34–0.48) |
| Micronesia  (Federated States of) | 0.01(0.01–0.01) | 19.47(17.57–21.58) | 0.01(0.01–0.01) | 19.83(17.61–22.02) | 42.5 | 0.14(0.11–0.17) |
| Monaco | 0.01(0.01–0.01) | 13.69(11.98–15.56) | 0.02(0.01–0.02) | 16.44(14.59–18.93) | 58.51 | 0.71(0.68–0.73) |
| Mongolia | 0.1(0.09–0.11) | 10.22(9.07–11.29) | 0.16(0.14–0.19) | 8.39(7.33–9.52) | 63.41 | −0.75(−0.81–−0.7) |
| Montenegro | 0.07(0.06–0.08) | 11.87(10.49–13.34) | 0.13(0.12–0.15) | 13.14(11.64–14.76) | 87.46 | 0.44(0.36–0.51) |
| Morocco | 0.99(0.86–1.12) | 8.13(7.14–9.11) | 2.73(2.42–3.05) | 9.84(8.84–10.91) | 176.54 | 0.76(0.73–0.8) |
| Mozambique | 0.38(0.33–0.45) | 7.79(6.88–8.91) | 0.75(0.66–0.86) | 8.27(7.29–9.29) | 97.29 | 0.21(0.19–0.23) |
| Myanmar | 2.32(2.01–2.63) | 11.46(10.22–12.81) | 5.33(4.69–5.99) | 12.73(11.36–14.25) | 129.45 | 0.43(0.39–0.47) |
| Namibia | 0.05(0.05–0.06) | 8.47(7.63–9.44) | 0.12(0.11–0.13) | 9.5(8.49–10.58) | 121.23 | 0.36(0.31–0.42) |
| Nauru | 0(0–0) | 20.53(18.28–22.93) | 0(0–0) | 19.44(17.41–22.01) | −3.27 | 0(−0.07–0.06) |
| Nepal | 0.58(0.51–0.67) | 7.5(6.72–8.39) | 1.75(1.54–1.96) | 8.69(7.78–9.61) | 200.94 | 0.53(0.49–0.58) |
| Netherlands | 3.2(2.94–3.47) | 15.42(14.08–16.76) | 5.29(4.74–5.85) | 14.97(13.39–16.63) | 65.61 | −0.28(−0.42–−0.15) |
| New Zealand | 0.49(0.4–0.58) | 12.19(10.16–14.31) | 1.09(0.9–1.29) | 13.79(11.42–16.18) | 124.41 | 0.54(0.48–0.6) |
| Nicaragua | 0.11(0.1–0.13) | 8.05(7.14–9.07) | 0.38(0.33–0.44) | 9.2(8.21–10.53) | 233.16 | 0.44(0.42–0.47) |
| Niger | 0.2(0.17–0.23) | 9.49(8.52–10.73) | 0.61(0.53–0.69) | 10.13(9.08–11.25) | 205.16 | 0.29(0.22–0.36) |
| Nigeria | 3.44(2.87–4.05) | 9.47(7.87–11.13) | 7.17(6.02–8.37) | 10.32(8.55–12.14) | 108.19 | 0.33(0.26–0.4) |
| Niue | 0(0–0) | 19.26(17.02–21.86) | 0(0–0) | 19.19(16.86–21.53) | −6.81 | 0.09(0.04–0.13) |
| North Macedonia | 0.21(0.19–0.23) | 12.19(11.06–13.32) | 0.43(0.38–0.48) | 13.75(12.51–15.22) | 104.85 | 0.46(0.42–0.49) |
| Northern Mariana Islands | 0(0–0) | 23.46(20.77–26.6) | 0.01(0.01–0.01) | 20.75(18.27–23.3) | 196.3 | −0.39(−0.44–−0.34) |
| Norway | 0.58(0.48–0.7) | 8.17(6.88–9.57) | 1.32(1.1–1.56) | 13.62(11.39–16.04) | 125.71 | 2.14(2–2.29) |
| Oman | 0.07(0.06–0.08) | 13.21(11.55–14.86) | 0.21(0.19–0.24) | 17.61(15.55–20.08) | 222.34 | 1.14(1.05–1.23) |
| Pakistan | 4.57(3.81–5.35) | 8.84(7.34–10.3) | 8.93(7.53–10.43) | 9.81(8.25–11.46) | 95.53 | 0.36(0.31–0.41) |
| Palau | 0(0–0) | 21.77(19.48–24.47) | 0(0–0) | 20.65(18.35–23.3) | 91.7 | −0.13(−0.19–−0.08) |
| Palestine | 0.08(0.07–0.09) | 10.86(9.64–12.26) | 0.22(0.19–0.24) | 11.28(10.06–12.71) | 160.17 | 0.21(0.14–0.28) |
| Panama | 0.14(0.13–0.16) | 10.07(8.94–11.4) | 0.51(0.45–0.57) | 12.4(10.96–13.77) | 256.34 | 0.6(0.55–0.65) |
| Papua New Guinea | 0.22(0.19–0.25) | 14.36(12.68–16.2) | 0.52(0.45–0.6) | 13.33(11.84–14.96) | 138.89 | −0.2(−0.23–−0.16) |
| Paraguay | 0.19(0.17–0.21) | 9.3(8.36–10.31) | 0.57(0.5–0.63) | 10.65(9.49–11.82) | 191.23 | 0.43(0.37–0.49) |
| Peru | 1.17(1.04–1.3) | 10.53(9.46–11.67) | 3.96(3.58–4.35) | 12.47(11.26–13.72) | 238.37 | 0.58(0.51–0.65) |
| Philippines | 2.68(2.25–3.14) | 10.42(8.75–12.21) | 7.87(6.6–9.17) | 11.29(9.49–13.22) | 193.45 | 0.37(0.32–0.43) |
| Poland | 5.7(4.69–6.78) | 12.99(10.74–15.38) | 9.16(7.93–10.42) | 12.66(11–14.37) | 60.76 | −0.14(−0.18–−0.11) |
| Portugal | 1.5(1.31–1.71) | 10.52(9.25–11.88) | 3.22(2.87–3.62) | 12.82(11.47–14.43) | 115.37 | 0.7(0.66–0.74) |
| Puerto Rico | 0.4(0.36–0.45) | 10.86(9.76–12.13) | 0.87(0.76–0.98) | 11.52(10.03–13.13) | 117.75 | −0.02(−0.09–0.04) |
| Qatar | 0.01(0.01–0.01) | 14.53(12.83–16.29) | 0.11(0.09–0.13) | 18.38(16.31–20.69) | 796.51 | 1.01(0.9–1.12) |
| Republic of Korea | 2.03(1.78–2.33) | 7.8(6.95–8.77) | 9.06(8.13–10.37) | 10.14(9.13–11.51) | 346.78 | 0.95(0.88–1.01) |
| Republic of Moldova | 0.46(0.4–0.53) | 11.1(9.87–12.53) | 0.47(0.41–0.53) | 8.06(7.13–9.08) | 1.83 | −1.26(−1.33–−1.19) |
| Romania | 2.67(2.39–3.01) | 9.68(8.77–10.82) | 4.3(3.93–4.89) | 10.98(10.02–12.43) | 61.14 | 0.47(0.44–0.5) |
| Russian Federation | 18.02(14.81–21.49) | 10.27(8.55–12.11) | 24.23(20.03–28.6) | 10.16(8.47–11.93) | 34.43 | −0.04(−0.12–0.04) |
| Rwanda | 0.19(0.17–0.22) | 7.88(6.98–8.96) | 0.4(0.34–0.46) | 7.91(6.97–9) | 103.54 | 0.07(0.03–0.11) |
| Saint Kitts and Nevis | 0.01(0.01–0.01) | 14.75(13.45–16.22) | 0.01(0.01–0.01) | 15.1(13.46–17.21) | 46.75 | −0.06(−0.13–0) |
| Saint Lucia | 0.01(0.01–0.01) | 12.49(11.3–13.73) | 0.03(0.03–0.03) | 13.62(12.1–15.15) | 169.35 | 0.15(0.07–0.23) |
| Saint Vincent and the Grenadines | 0.01(0.01–0.01) | 10.23(9.06–11.46) | 0.02(0.01–0.02) | 12.01(10.67–13.38) | 113.71 | 0.55(0.44–0.65) |
| Samoa | 0.01(0.01–0.02) | 18.19(16.23–20.26) | 0.02(0.02–0.03) | 17.74(16.14–19.57) | 62.09 | 0(−0.03–0.03) |
| San Marino | 0(0–0) | 11.08(9.9–12.71) | 0.01(0.01–0.01) | 11.26(9.9–12.94) | 93.95 | −0.02(−0.06–0.02) |
| Sao Tome and Principe | 0.01(0–0.01) | 9.65(8.57–10.87) | 0.01(0.01–0.01) | 10.92(9.7–12.32) | 74.61 | 0.53(0.46–0.6) |
| Saudi Arabia | 0.6(0.53–0.68) | 12.63(11.16–14.19) | 1.95(1.71–2.22) | 16(14.38–18.2) | 224.72 | 0.81(0.77–0.85) |
| Senegal | 0.27(0.24–0.31) | 10.17(9.11–11.33) | 0.72(0.63–0.8) | 11.4(10.21–12.56) | 164.54 | 0.46(0.38–0.53) |
| Serbia | 1.15(1.02–1.29) | 10.81(9.79–11.81) | 1.94(1.75–2.19) | 11.49(10.4–12.8) | 68.68 | 0.21(0.19–0.23) |
| Seychelles | 0.01(0.01–0.01) | 15.76(14.15–17.56) | 0.02(0.02–0.02) | 17.24(15.24–19.59) | 91 | 0.4(0.34–0.46) |
| Sierra Leone | 0.16(0.14–0.17) | 9.05(8.16–10.02) | 0.32(0.28–0.36) | 10.51(9.53–11.68) | 102.14 | 0.58(0.53–0.64) |
| Singapore | 0.17(0.16–0.19) | 8.6(7.74–9.42) | 0.7(0.62–0.79) | 9.29(8.36–10.4) | 298.94 | 0.34(0.28–0.4) |
| Slovakia | 0.61(0.54–0.68) | 10.02(9.02–11.15) | 0.95(0.83–1.08) | 9.98(8.85–11.29) | 56.04 | −0.05(−0.08–−0.01) |
| Slovenia | 0.28(0.25–0.31) | 11.32(10.11–12.57) | 0.49(0.45–0.54) | 11.08(9.97–12.36) | 77.51 | −0.16(−0.2–−0.11) |
| Solomon Islands | 0.02(0.02–0.02) | 17.85(15.97–20.06) | 0.04(0.04–0.05) | 17.6(15.72–19.57) | 121.29 | 0(−0.02–0.03) |
| Somalia | 0.14(0.12–0.17) | 7.23(6.33–8.21) | 0.38(0.33–0.44) | 7.15(6.28–8.09) | 163.63 | 0.01(−0.03–0.05) |
| South Africa | 1.44(1.21–1.68) | 7.73(6.45–9.01) | 3.65(3.04–4.28) | 9.22(7.67–10.79) | 152.61 | 0.66(0.6–0.73) |
| South Sudan | 0.17(0.15–0.2) | 8.1(7.15–9.23) | 0.25(0.21–0.29) | 7.86(6.91–8.98) | 46.87 | −0.1(−0.13–−0.07) |
| Spain | 8.12(7.38–8.97) | 14.22(13–15.6) | 14.45(12.77–16.25) | 14.61(12.68–16.66) | 77.84 | −0.04(−0.12–0.04) |
| Sri Lanka | 1.18(1.01–1.36) | 12.09(10.52–13.96) | 3.32(2.84–3.83) | 13.41(11.66–15.37) | 182.55 | 0.4(0.34–0.46) |
| Sudan | 0.8(0.7–0.89) | 9.53(8.54–10.56) | 1.55(1.38–1.74) | 9.42(8.45–10.62) | 94.71 | −0.03(−0.06–0.01) |
| Suriname | 0.02(0.02–0.02) | 8.68(7.7–9.87) | 0.06(0.05–0.07) | 10.63(9.56–11.76) | 185.5 | 0.65(0.52–0.78) |
| Sweden | 1.96(1.6–2.36) | 12.39(10.23–14.67) | 3.19(2.6–3.83) | 14.56(12.05–17.22) | 62.82 | 0.69(0.62–0.75) |
| Switzerland | 1.46(1.3–1.63) | 13.38(11.88–14.93) | 2.45(2.19–2.71) | 13.7(12.22–15.2) | 67.45 | 0.29(0.21–0.37) |
| Syrian Arab Republic | 0.46(0.41–0.52) | 10.61(9.53–11.83) | 1.18(1.03–1.33) | 11.58(10.33–12.93) | 153.67 | 0.39(0.31–0.47) |
| Taiwan  (Province of China) | 2.21(1.95–2.5) | 14.91(13.17–16.65) | 6.78(5.85–7.44) | 17(14.79–18.61) | 207.38 | 0.92(0.61–1.24) |
| Tajikistan | 0.28(0.24–0.33) | 10.83(9.19–12.65) | 0.56(0.49–0.65) | 16.59(14.74–18.62) | 100.24 | 1.61(1.52–1.71) |
| Thailand | 4.03(3.51–4.63) | 13.09(11.49–14.89) | 11.95(10.47–13.8) | 11.94(10.49–13.76) | 196.3 | −0.23(−0.3–−0.15) |
| Timor–Leste | 0.02(0.02–0.03) | 10.13(8.98–11.52) | 0.08(0.07–0.09) | 10.99(9.77–12.39) | 267.76 | 0.42(0.36–0.49) |
| Togo | 0.1(0.08–0.11) | 9.86(8.89–10.9) | 0.3(0.26–0.34) | 10.66(9.56–11.88) | 209.24 | 0.3(0.24–0.37) |
| Tokelau | 0(0–0) | 17.54(15.57–19.63) | 0(0–0) | 17.47(15.43–19.62) | −8.34 | 0.07(0.04–0.1) |
| Tonga | 0.01(0.01–0.01) | 16.67(14.79–18.81) | 0.01(0.01–0.01) | 15.57(13.85–17.35) | 43.87 | −0.12(−0.16–−0.08) |
| Trinidad and Tobago | 0.08(0.07–0.09) | 10.02(9.03–11.26) | 0.19(0.17–0.22) | 10.67(9.49–12.04) | 140.52 | 0.07(0–0.14) |
| Tunisia | 0.42(0.36–0.47) | 9.35(8.29–10.54) | 1.23(1.1–1.39) | 10.52(9.37–11.83) | 195.94 | 0.43(0.41–0.44) |
| Turkey | 3.18(2.77–3.62) | 10.25(8.92–11.62) | 10.21(8.95–11.5) | 12.3(10.75–13.82) | 220.81 | 0.78(0.71–0.86) |
| Turkmenistan | 0.14(0.12–0.16) | 8.14(7.27–9.07) | 0.31(0.27–0.35) | 8.85(7.86–9.95) | 118.28 | 0.26(0.23–0.29) |
| Tuvalu | 0(0–0) | 16.38(14.67–18.25) | 0(0–0) | 17.64(15.78–19.72) | 67.4 | 0.35(0.3–0.39) |
| Uganda | 0.44(0.37–0.5) | 7.8(6.81–8.85) | 0.93(0.81–1.07) | 7.81(6.88–8.86) | 114.11 | 0.02(−0.01–0.05) |
| Ukraine | 7.66(6.31–9.12) | 10.72(8.93–12.6) | 8.57(7.11–10.15) | 10.99(9.19–12.88) | 11.92 | 0.06(0.03–0.09) |
| United Arab Emirates | 0.05(0.04–0.06) | 16.65(14.91–18.6) | 0.47(0.4–0.56) | 19.02(17.12–21.18) | 854.71 | 0.58(0.49–0.66) |
| United Kingdom | 13.85(11.69–16.29) | 14.8(12.65–17.12) | 19.27(16.38–22.22) | 14.84(12.69–17.09) | 39.14 | −0.1(−0.18–−0.01) |
| United Republic of Tanzania | 0.69(0.6–0.79) | 7.44(6.56–8.33) | 1.66(1.5–1.85) | 7.77(7.08–8.63) | 138.62 | 0.19(0.16–0.22) |
| United States of America | 37.73(31.15–44.53) | 11.32(9.45–13.31) | 153.61(132.41–176.76) | 26.44(22.82–30.4) | 307.09 | 2.87(2.35–3.38) |
| United States Virgin Islands | 0.01(0.01–0.01) | 15.46(14.02–16.89) | 0.03(0.03–0.04) | 16.89(15.16–19.01) | 176.7 | 0.29(0.21–0.37) |
| Uruguay | 0.47(0.43–0.54) | 11.58(10.53–13.13) | 0.68(0.61–0.76) | 11.86(10.7–13.37) | 44.2 | 0.25(0.18–0.31) |
| Uzbekistan | 1.04(0.9–1.18) | 10.02(8.79–11.39) | 2(1.73–2.33) | 13.33(12.05–14.63) | 93.3 | 1(0.94–1.06) |
| Vanuatu | 0.01(0.01–0.01) | 19.6(17.56–22.1) | 0.03(0.02–0.03) | 18.69(16.98–20.59) | 160.1 | −0.07(−0.11–−0.02) |
| Venezuela | 0.82(0.72–0.92) | 9.17(8.14–10.25) | 2.99(2.68–3.32) | 10.82(9.68–12.18) | 266.08 | 0.51(0.47–0.54) |
| Viet Nam | 4.13(3.63–4.65) | 10.91(9.67–12.25) | 10.83(9.55–12.53) | 12.87(11.43–14.72) | 162.46 | 0.72(0.64–0.79) |
| Yemen | 0.33(0.28–0.37) | 7.9(6.95–8.9) | 1(0.88–1.13) | 8.81(7.81–9.84) | 207.77 | 0.52(0.47–0.56) |
| Zambia | 0.21(0.18–0.24) | 8.97(8.01–10.07) | 0.49(0.43–0.55) | 8.89(7.93–9.86) | 132.04 | −0.06(−0.09–−0.02) |
| Zimbabwe | 0.31(0.27–0.35) | 9.44(8.53–10.51) | 0.53(0.47–0.58) | 9.6(8.75–10.52) | 70.39 | −0.02(−0.09–0.04) |

EAPC: estimated annual percentage change; ASR, age-standardized rate; CI, confidence interval; UI: uncertainty interval.

**Supplementary table 3**. The percentage changes and EAPCs of Parkinson’s disease prevalence at the national level from 1990 to 2019.

|  | **1990** | | **2019** | | **1990-2019** | |
| --- | --- | --- | --- | --- | --- | --- |
| **Characteristics** | Number  ×10^3^ (95% UI) | ASR/100,000  (95% UI) | Number  ×10^3^ (95% UI) | ASR/100,000  (95% UI) | Percentage change (%) | EAPC  (95%CI) |
| Afghanistan | 4.54(3.67–5.6) | 71.66(57.94–87.5) | 7.35(6.14–8.81) | 69.27(58.17–82.42) | 61.79 | −0.05(−0.12–0.02) |
| Albania | 1.66(1.4–1.97) | 89.9(75.31–106.42) | 4.35(3.6–5.16) | 97.92(81.37–115.95) | 162.45 | 0.27(0.24–0.30) |
| Algeria | 7.55(6.25–9.11) | 72.93(60.85–87.2) | 23.96(19.89–28.84) | 80.36(66.55–96.82) | 217.43 | 0.37(0.32–0.41) |
| American Samoa | 0.03(0.02–0.03) | 144.79(120.8–174.09) | 0.06(0.05–0.07) | 138.05(112.94–162.56) | 122.97 | −0.02(−0.09–0.06) |
| Andorra | 0.05(0.05–0.07) | 107.12(89.48–128.21) | 0.16(0.13–0.2) | 114.78(94.95–138.35) | 195.83 | 0.25(0.22–0.29) |
| Angola | 1.67(1.33–2.04) | 53.59(43.5–64.67) | 5.25(4.27–6.41) | 59.06(47.88–71.66) | 215.13 | 0.44(0.4–0.48) |
| Antigua and Barbuda | 0.05(0.04–0.06) | 87.14(72.82–102.27) | 0.1(0.08–0.12) | 104.59(86.2–125.35) | 99.54 | 0.55(0.41–0.69) |
| Argentina | 28.24(24.15–33.71) | 90.43(77.4–107.68) | 47.98(40.71–57.8) | 86.44(73.27–103.91) | 69.91 | 0(−0.11–0.12) |
| Armenia | 1.75(1.46–2.06) | 75.27(62.62–87.03) | 3.3(2.67–3.92) | 80.42(65.01–95.5) | 87.97 | 0.21(0.14–0.29) |
| Australia | 26.33(22.62–31.02) | 133.61(114.97–156.88) | 60.45(48.26–74.14) | 138.71(111.15–170.13) | 129.58 | −0.15(−0.46–0.15) |
| Austria | 13.55(11.68–15.89) | 107.58(92.72–126.65) | 24.83(20.76–29.17) | 128.32(107.36–150.65) | 83.27 | 0.46(0.38–0.54) |
| Azerbaijan | 3.24(2.72–3.83) | 74.56(62.22–88.07) | 6.46(5.36–7.75) | 88.62(74.38–104.51) | 99.23 | 0.5(0.46–0.54) |
| Bahamas | 0.12(0.1–0.15) | 87.5(73.43–103.77) | 0.34(0.29–0.41) | 96.18(81.79–114.2) | 174.19 | 0.18(0.09–0.27) |
| Bahrain | 0.11(0.09–0.14) | 83.54(67.85–99.85) | 0.67(0.55–0.83) | 94(75.92–113.37) | 493.12 | 0.48(0.43–0.53) |
| Bangladesh | 27.2(22.35–33) | 65.77(53.51–79.69) | 81.65(66.48–99.57) | 66.86(54.78–81.17) | 200.2 | −0.08(−0.12–−0.03) |
| Barbados | 0.26(0.22–0.3) | 82.11(70.96–96.16) | 0.5(0.43–0.59) | 100.35(86.07–118.02) | 92.3 | 0.52(0.42–0.62) |
| Belarus | 10.46(8.57–12.75) | 82.13(67.49–99.74) | 13.73(11.59–16.9) | 84.82(71.54–104.19) | 31.27 | −0.02(−0.07–0.03) |
| Belgium | 17.55(15.26–20.9) | 108.81(94.71–129.05) | 29.84(24.13–35.37) | 121.84(98.66–144.65) | 70.1 | 0.65(0.59–0.71) |
| Belize | 0.07(0.06–0.09) | 80.6(66.17–96.85) | 0.23(0.19–0.27) | 91.98(77.32–108.93) | 215.29 | 0.34(0.2–0.47) |
| Benin | 1.16(0.95–1.39) | 65.03(53.17–77.06) | 2.91(2.42–3.49) | 71.95(59.22–86.12) | 150.77 | 0.35(0.22–0.47) |
| Bermuda | 0.06(0.05–0.08) | 108.48(91.01–129.12) | 0.16(0.14–0.19) | 114.91(99.15–139.61) | 143.05 | 0.15(0.03–0.28) |
| Bhutan | 0.12(0.1–0.15) | 61.46(50.45–74.1) | 0.38(0.32–0.45) | 73.7(61.19–86.97) | 205.78 | 0.57(0.53–0.61) |
| Bolivia | 2.48(2.07–2.89) | 83.54(69.87–96.07) | 7.59(6.28–8.92) | 92.23(76.18–108.54) | 206.36 | 0.25(0.18–0.31) |
| Bosnia and Herzegovina | 3.25(2.71–3.87) | 91.61(75.88–108.12) | 5.66(4.6–6.85) | 92.47(75.73–111.58) | 74.09 | 0.07(0.04–0.11) |
| Botswana | 0.26(0.21–0.31) | 54.37(45.64–64.52) | 0.77(0.63–0.91) | 70.49(58.1–82.43) | 200.05 | 0.81(0.75–0.88) |
| Brazil | 58.57(48.57–69.38) | 73.42(60.72–87.18) | 200.71(166.59–238.31) | 87.52(72.42–103.94) | 242.66 | 0.63(0.54–0.73) |
| Brunei Darussalam | 0.06(0.05–0.08) | 76.98(62.42–94.63) | 0.22(0.18–0.27) | 91.64(73.73–110.41) | 258.69 | 0.71(0.62–0.8) |
| Bulgaria | 11.92(10.08–13.79) | 99.37(85.2–114.42) | 14.48(11.93–17.61) | 90.92(75–110.14) | 21.44 | −0.64(−0.76–−0.51) |
| Burkina Faso | 2.21(1.77–2.63) | 61.83(50.73–73.28) | 5.12(4.17–6.26) | 69.28(56.55–83.87) | 132.34 | 0.45(0.32–0.57) |
| Burundi | 1.11(0.91–1.37) | 53.23(43.69–64.41) | 2.1(1.71–2.58) | 56.04(45.47–67.91) | 88.57 | 0.24(0.18–0.3) |
| Cabo Verde | 0.14(0.12–0.17) | 59.33(48.04–71.4) | 0.3(0.25–0.35) | 73.86(61.89–85.58) | 108 | 0.84(0.75–0.93) |
| Cambodia | 3.14(2.56–3.76) | 83.4(67.88–99.79) | 9.35(7.8–11.04) | 89.57(74.99–105.4) | 198.31 | 0.32(0.22–0.41) |
| Cameroon | 2.48(2.07–2.92) | 70(58.77–81.84) | 7.62(6.39–9.11) | 80.9(67.45–95.11) | 207.45 | 0.56(0.44–0.69) |
| Canada | 26.45(22.52–31.17) | 80.49(68.74–94.82) | 87.22(70.61–107.43) | 120.84(98.09–148.53) | 229.83 | 2.14(1.49–2.8) |
| Central African Republic | 0.49(0.4–0.61) | 52.78(42.48–64.61) | 0.91(0.74–1.11) | 53.02(43.26–64.23) | 84.65 | 0.11(0.07–0.14) |
| Chad | 1.52(1.23–1.82) | 60.03(49.03–71.63) | 3.11(2.58–3.73) | 67.56(55.85–80.75) | 104.96 | 0.43(0.35–0.52) |
| Chile | 7.78(6.72–9.1) | 84.61(73.01–98.52) | 22.47(18.16–27.33) | 93.21(75.42–113.5) | 188.97 | 0.65(0.55–0.75) |
| China | 791.41(650.62–948.09) | 107.35(88.29–127.64) | 2848.83(2336.62–3425.74) | 145.83(120.65–173.79) | 259.97 | 1.01(0.74–1.29) |
| Colombia | 11.59(9.8–13.49) | 73.88(62.89–85.48) | 45.64(38.59–54.53) | 85.65(72.1–102.33) | 293.9 | 0.46(0.37–0.55) |
| Comoros | 0.12(0.1–0.14) | 57.1(47.07–68.71) | 0.27(0.22–0.33) | 59.94(49.57–72.43) | 132.33 | 0.19(0.17–0.21) |
| Congo | 0.52(0.43–0.63) | 58.28(47.8–69.12) | 1.37(1.12–1.65) | 64.05(52.65–76.44) | 163.52 | 0.43(0.38–0.48) |
| Cook Islands | 0.02(0.01–0.02) | 152.17(124.75–181.31) | 0.04(0.03–0.04) | 143.81(117.08–171.78) | 109.05 | −0.04(−0.11–0.02) |
| Costa Rica | 1.42(1.17–1.67) | 85.57(70.46–100.92) | 4.9(4.09–5.75) | 97.33(81.06–114.27) | 245.87 | 0.41(0.37–0.46) |
| Croatia | 5.4(4.42–6.59) | 87.24(71.09–106.15) | 9.26(7.65–10.98) | 96.71(80.32–114.74) | 71.36 | 0.26(0.21–0.31) |
| Cuba | 7.53(6.36–8.81) | 73.47(62.46–85.73) | 18.28(15.47–21.69) | 93.48(79.41–111.58) | 142.66 | 0.95(0.83–1.06) |
| Cyprus | 1(0.79–1.24) | 123.9(99.35–150.49) | 2.5(2.04–3) | 126.35(103.81–151.12) | 149.53 | 0.1(0.08–0.11) |
| Czechia | 11.36(9.38–13.94) | 80.27(66.4–98.39) | 19.9(16.44–23.24) | 88.4(73.17–102.94) | 75.18 | 0.24(0.17–0.31) |
| Côte d'Ivoire | 2.09(1.73–2.54) | 72.29(60.23–86.07) | 6.37(5.21–7.77) | 78.77(64.2–94.9) | 204.53 | 0.35(0.25–0.45) |
| Democratic People's Republic of Korea | 15.27(12.49–18.27) | 112.36(91.26–134.34) | 31.5(25.6–37.4) | 101.75(82.52–120.55) | 106.33 | −0.35(−0.41–−0.28) |
| Democratic Republic of the Congo | 6.95(5.65–8.4) | 55.24(45.29–66.49) | 16.98(13.64–20.98) | 57.35(46.41–69.59) | 144.17 | 0.14(0.06–0.22) |
| Denmark | 7.74(6.51–9) | 90.17(75.85–104.94) | 13.97(11.69–16.28) | 115.22(97.06–134.54) | 80.63 | 1.12(1.04–1.21) |
| Djibouti | 0.06(0.05–0.07) | 54.84(44.46–66.66) | 0.29(0.23–0.35) | 60.69(48.88–73.61) | 381.83 | 0.4(0.35–0.44) |
| Dominica | 0.07(0.06–0.08) | 87.53(74.03–104.05) | 0.09(0.07–0.11) | 95.04(79.64–114.45) | 30.83 | 0.17(0.02–0.32) |
| Dominican Republic | 2.32(1.93–2.77) | 68.01(57.21–80.76) | 7.77(6.49–9.01) | 87.77(73.61–101.79) | 234.41 | 0.72(0.63–0.82) |
| Ecuador | 3.67(3.03–4.31) | 73.23(60.59–85.85) | 14.74(12.21–17.23) | 102.1(84.6–119.12) | 302.04 | 1.11(1.01–1.21) |
| Egypt | 17.62(14.41–21.12) | 71.9(58.75–85.9) | 43.84(36.13–52.6) | 84.36(69.12–101) | 148.76 | 0.5(0.47–0.53) |
| El Salvador | 2.06(1.74–2.38) | 73.43(61.73–84.6) | 5.58(4.72–6.46) | 91.4(77.05–106.16) | 170.52 | 0.7(0.6–0.8) |
| Equatorial Guinea | 0.09(0.07–0.1) | 52.38(42.8–62.31) | 0.27(0.22–0.32) | 67.02(54–80.04) | 211.38 | 1.04(0.98–1.11) |
| Eritrea | 0.39(0.32–0.48) | 50.32(41.38–61.17) | 1.25(1.02–1.52) | 57.93(47.43–70.03) | 220.39 | 0.44(0.42–0.46) |
| Estonia | 2.01(1.69–2.43) | 96.96(81.81–117.32) | 2.62(2.17–3.19) | 91.65(76.55–111.28) | 30.42 | −0.29(−0.36–−0.23) |
| Eswatini | 0.14(0.11–0.17) | 56.41(46.42–66.87) | 0.31(0.26–0.37) | 63.96(53.03–75.65) | 122.64 | 0.28(0.19–0.36) |
| Ethiopia | 8.94(7.43–10.75) | 53.77(44–64.03) | 20.4(16.9–24.5) | 55.68(45.84–66.76) | 128.14 | 0.11(0.07–0.15) |
| Fiji | 0.4(0.33–0.49) | 141.32(114.17–170.42) | 0.82(0.66–1) | 130.64(106.09–156.41) | 105.76 | −0.13(−0.19–−0.07) |
| Finland | 7.62(6.19–8.91) | 103.76(84.53–120.82) | 16.93(13.89–21.22) | 128.86(106.66–161.79) | 122.07 | 0.72(0.67–0.77) |
| France | 91.17(77.82–106.36) | 103.34(87.87–120.47) | 177.99(155.1–209.11) | 120.34(104.52–142.32) | 95.23 | 0.7(0.6–0.81) |
| Gabon | 0.33(0.27–0.39) | 65.86(53.95–78.45) | 0.65(0.55–0.77) | 73(61.64–86.87) | 99.49 | 0.41(0.37–0.46) |
| Gambia | 0.19(0.15–0.22) | 64.36(53.55–76.72) | 0.64(0.53–0.75) | 75.67(63.07–89.94) | 243.68 | 0.53(0.43–0.63) |
| Georgia | 4.72(3.83–5.74) | 81.16(66.16–98.61) | 4.66(3.88–5.46) | 74.61(62.31–87.43) | −1.32 | −0.49(−0.55–−0.42) |
| Germany | 134.48(113.5–157.03) | 101.58(85.69–118.17) | 272.98(218.73–324.62) | 130.49(105.87–155.19) | 102.98 | 1.68(1.33–2.03) |
| Ghana | 3.01(2.49–3.62) | 61.43(51.38–74.31) | 9.17(7.65–10.81) | 70.17(58.62–82.32) | 205.17 | 0.46(0.32–0.6) |
| Greece | 16.62(13.81–19.32) | 106.76(88.87–123.5) | 30.35(25.54–36.19) | 115.88(98.56–137.39) | 82.59 | 0.3(0.25–0.34) |
| Greenland | 0.03(0.02–0.03) | 101.3(80.69–124.37) | 0.07(0.05–0.08) | 110.96(91.25–135.51) | 149.38 | 0.33(0.31–0.36) |
| Grenada | 0.06(0.05–0.07) | 75.87(65.25–89) | 0.1(0.08–0.12) | 95.21(81.32–119.11) | 67.67 | 0.62(0.54–0.7) |
| Guam | 0.09(0.07–0.11) | 147.97(121.2–174.61) | 0.23(0.19–0.27) | 124.55(102.39–147.83) | 156.34 | −0.6(−0.67–−0.53) |
| Guatemala | 2.2(1.8–2.65) | 71.92(58.86–85.88) | 8.47(7.06–9.92) | 79.45(66.36–92.73) | 284.38 | 0.2(0.11–0.29) |
| Guinea | 1.83(1.47–2.19) | 63.18(51.46–75.9) | 3.52(2.92–4.26) | 74.26(61.82–89.76) | 92.71 | 0.56(0.44–0.69) |
| Guinea–Bissau | 0.22(0.17–0.26) | 65.6(53.32–78.79) | 0.4(0.33–0.47) | 70.24(58.93–82.52) | 83.36 | 0.26(0.16–0.35) |
| Guyana | 0.27(0.22–0.33) | 79.49(66.55–97.41) | 0.49(0.42–0.58) | 88.73(77.29–104.54) | 81.2 | 0.29(0.15–0.42) |
| Haiti | 1.92(1.55–2.31) | 68.05(55.43–81.55) | 4.85(3.96–5.8) | 81.23(66.98–96.83) | 153.2 | 0.53(0.44–0.63) |
| Honduras | 1.41(1.18–1.66) | 75.09(63.31–88.18) | 4.85(3.99–5.67) | 87.86(72.19–103.15) | 245.28 | 0.44(0.38–0.5) |
| Hungary | 11.64(9.91–13.82) | 77.25(65.93–91.58) | 17.33(14.58–20.57) | 83.81(70.7–99.13) | 48.86 | 0.33(0.23–0.43) |
| Iceland | 0.38(0.32–0.44) | 127.24(106.54–148.24) | 0.85(0.7–1.03) | 147.54(121.47–179.34) | 125.31 | 0.57(0.51–0.62) |
| India | 230.28(190.52–277.45) | 61.97(50.82–73.64) | 770.78(635.13–919.36) | 73.9(60.86–87.98) | 234.71 | 0.59(0.55–0.63) |
| Indonesia | 68.1(56.76–81.45) | 84.16(69.5–100.36) | 165.62(137.61–197.28) | 89.93(74.09–107.26) | 143.21 | 0.21(0.14–0.27) |
| Iran  (Islamic Republic of) | 15.66(13.07–18.7) | 75.29(62.56–89.58) | 56.51(47.33–66.92) | 85.12(70.23–101.35) | 260.86 | 0.39(0.34–0.45) |
| Iraq | 4.9(4.06–5.86) | 70.62(58.52–84.71) | 14.38(11.8–17.14) | 74.22(61.06–87.87) | 193.44 | 0.2(0.14–0.25) |
| Ireland | 4.65(3.9–5.48) | 110.63(92.42–129.72) | 10.13(8.44–12.49) | 131.58(110.06–161.75) | 117.63 | 0.66(0.6–0.71) |
| Israel | 5.74(4.83–6.79) | 115.86(97.71–136.72) | 13.8(11.56–16.42) | 115.53(96.97–137.27) | 140.22 | 0.07(−0.01–0.15) |
| Italy | 154.61(127.9–184.62) | 166.08(138.15–197.41) | 207.1(168.37–251.06) | 131.41(107.85–156.77) | 33.95 | −1.06(−1.35–−0.77) |
| Jamaica | 1.4(1.18–1.64) | 76.62(64.68–89.68) | 2.67(2.3–3.09) | 88.7(76.31–103.59) | 90.5 | 0.51(0.34–0.69) |
| Japan | 104.17(86.03–124.17) | 61.88(51.16–73.13) | 299.37(242.44–359.1) | 77.31(64.16–91.81) | 187.4 | 0.44(0.31–0.57) |
| Jordan | 0.79(0.66–0.93) | 74.73(62.23–87.32) | 4.13(3.38–4.88) | 76.95(63.38–91.51) | 425.73 | −0.05(−0.18–0.07) |
| Kazakhstan | 8.6(7.19–10.31) | 75.85(63.03–90.26) | 12.85(10.59–15.7) | 84.16(69.73–102.85) | 49.33 | 0.34(0.23–0.45) |
| Kenya | 3.9(3.23–4.67) | 54.25(44.43–64.85) | 10.59(8.81–12.61) | 56.35(46.47–67.27) | 171.5 | 0.13(0.1–0.16) |
| Kiribati | 0.04(0.03–0.04) | 119.43(97.89–146.07) | 0.06(0.05–0.07) | 112.87(92.69–135.89) | 70.2 | −0.1(−0.15–−0.06) |
| Kuwait | 0.38(0.31–0.46) | 78.01(64.12–93.83) | 1.58(1.29–1.92) | 71.37(59.07–86.13) | 315.08 | −0.31(−0.37–−0.25) |
| Kyrgyzstan | 1.94(1.59–2.31) | 67.78(55.08–81.37) | 2.55(2.13–3.01) | 64.49(54.11–75.58) | 31.34 | −0.33(−0.37–−0.28) |
| Lao People's Democratic Republic | 1.43(1.17–1.7) | 81.51(67.33–97.66) | 3.32(2.77–3.97) | 89.59(74.54–106.36) | 131.72 | 0.28(0.2–0.37) |
| Latvia | 3.21(2.72–3.83) | 88.45(74.93–105.37) | 3.71(3.09–4.47) | 85.52(71.29–103.03) | 15.39 | −0.2(−0.27–−0.12) |
| Lebanon | 1.39(1.15–1.64) | 71.02(58.77–83.26) | 4.06(3.48–4.76) | 77.62(66.46–90.88) | 192.11 | 0.42(0.37–0.47) |
| Lesotho | 0.43(0.36–0.52) | 50.52(41.89–60.09) | 0.59(0.49–0.7) | 55.8(47.05–64.66) | 35.64 | 0.16(0.07–0.25) |
| Liberia | 0.63(0.51–0.75) | 64.48(52.66–77.01) | 1.17(0.96–1.4) | 70.61(57.98–84.31) | 86.71 | 0.32(0.18–0.46) |
| Libya | 1.22(1.02–1.45) | 74.1(61.48–87.39) | 3.83(3.18–4.45) | 84.07(69.28–98.34) | 213.54 | 0.6(0.53–0.67) |
| Lithuania | 3.66(3.09–4.42) | 80.97(68.29–97.65) | 5.31(4.52–6.26) | 85.45(72.25–100.96) | 44.91 | 0.24(0.19–0.3) |
| Luxembourg | 0.67(0.57–0.8) | 117.64(99.93–140.39) | 1.4(1.17–1.65) | 135.5(112.3–159.89) | 110.61 | 0.44(0.33–0.55) |
| Madagascar | 2.21(1.78–2.7) | 49.64(40.34–59.98) | 4.62(3.72–5.71) | 52.25(42.03–63.86) | 108.99 | 0.17(0.13–0.21) |
| Malawi | 1.78(1.45–2.17) | 55.12(45.51–66.83) | 3.57(2.9–4.29) | 56.77(46.34–68.1) | 100.96 | 0.13(0.11–0.16) |
| Malaysia | 7.73(6.23–9.52) | 97.09(77.61–118.89) | 25.91(20.54–31.51) | 107.99(85.56–131.74) | 234.98 | 0.39(0.32–0.47) |
| Maldives | 0.07(0.06–0.08) | 106.38(86.46–125.84) | 0.3(0.25–0.35) | 113.3(93.56–131.39) | 319.94 | 0.31(0.2–0.41) |
| Mali | 2(1.63–2.43) | 60.12(49.61–72.94) | 4.85(3.96–5.73) | 68.85(56.5–80.46) | 141.81 | 0.45(0.29–0.6) |
| Malta | 0.46(0.38–0.56) | 109.4(90.92–131.29) | 1.27(1.07–1.5) | 128.34(109.06–152) | 173.21 | 0.63(0.57–0.69) |
| Marshall Islands | 0.02(0.02–0.03) | 145.14(118.37–176.57) | 0.04(0.03–0.05) | 148.02(120.46–178.56) | 92.09 | 0.22(0.17–0.27) |
| Mauritania | 0.56(0.47–0.68) | 63.9(53.56–76.81) | 1.25(1.03–1.49) | 68.95(56.67–82.63) | 123.54 | 0.16(0.02–0.29) |
| Mauritius | 0.67(0.55–0.82) | 104.15(85.35–126.55) | 1.79(1.44–2.22) | 108.19(87.08–133.65) | 166.94 | 0.05(−0.03–0.14) |
| Mexico | 29.84(24.82–35.37) | 76.9(63.83–91.38) | 103.56(86.34–122.48) | 93.02(77.27–110.37) | 247.05 | 0.56(0.46–0.67) |
| Micronesia  (Federated States of) | 0.05(0.04–0.06) | 133.42(109.84–159.41) | 0.08(0.06–0.09) | 139.66(113.99–167.58) | 47.87 | 0.24(0.2–0.29) |
| Monaco | 0.09(0.08–0.11) | 119.71(99.14–142.65) | 0.15(0.12–0.18) | 142.99(119.13–172.15) | 57.91 | 0.68(0.66–0.71) |
| Mongolia | 0.7(0.57–0.84) | 73.27(59.49–87.34) | 1.24(1–1.51) | 66.41(52.87–80.46) | 77.14 | −0.4(−0.48–−0.31) |
| Montenegro | 0.54(0.44–0.65) | 92.03(75.43–110.67) | 0.99(0.8–1.19) | 98.62(80.65–117.79) | 83.13 | 0.31(0.21–0.41) |
| Morocco | 7.32(5.96–8.75) | 61.03(49.66–72.55) | 20.47(17.15–23.85) | 73.67(61.93–85.8) | 179.55 | 0.75(0.7–0.81) |
| Mozambique | 2.61(2.11–3.25) | 51.94(42.03–64.61) | 5.21(4.22–6.37) | 56.42(45.41–68.21) | 99.78 | 0.29(0.25–0.32) |
| Myanmar | 17.42(14.08–21.13) | 88.26(71.34–105.38) | 41.75(34.53–49.12) | 101.78(84.24–119.7) | 139.68 | 0.55(0.49–0.61) |
| Namibia | 0.37(0.3–0.44) | 57.71(48.42–69.28) | 0.85(0.71–1.02) | 68.23(57.34–81.61) | 131.43 | 0.51(0.42–0.61) |
| Nauru | 0(0–0) | 144.06(116.62–174.43) | 0(0–0) | 139.32(114.3–172.51) | −1.81 | 0.08(0–0.17) |
| Nepal | 4.16(3.37–5.1) | 52.87(43.03–64.44) | 12.94(10.55–15.47) | 64.17(52.81–75.8) | 210.9 | 0.67(0.62–0.71) |
| Netherlands | 27.59(23.77–31.27) | 132.34(113.82–150.08) | 46.14(39.26–53.35) | 128.42(108.75–149.15) | 67.26 | −0.32(−0.48–−0.16) |
| New Zealand | 4.38(3.57–5.26) | 109.64(89.82–131.07) | 10.21(8.33–12.3) | 126.27(103.61–151.46) | 133.35 | 0.59(0.5–0.69) |
| Nicaragua | 0.95(0.79–1.13) | 67.68(56.34–80.38) | 3.27(2.71–3.91) | 80.79(67.46–96.83) | 245.35 | 0.59(0.55–0.63) |
| Niger | 1.3(1.07–1.58) | 60.63(50.26–72.64) | 4(3.26–4.83) | 66.24(54.38–79.14) | 207.05 | 0.35(0.23–0.47) |
| Nigeria | 22.75(18.74–27.38) | 61.72(50.26–74.13) | 49.85(41.16–59.92) | 71.16(57.4–85.8) | 119.1 | 0.48(0.37–0.6) |
| Niue | 0(0–0) | 142.5(115.57–172.57) | 0(0–0) | 147.62(120.02–177.13) | −4.12 | 0.26(0.2–0.32) |
| North Macedonia | 1.46(1.22–1.7) | 87.39(72.99–101.54) | 3.02(2.49–3.58) | 97.44(81.19–115) | 107.02 | 0.42(0.37–0.47) |
| Northern Mariana Islands | 0.02(0.02–0.02) | 173.44(142.76–206.29) | 0.07(0.05–0.08) | 158.2(128.25–188.09) | 211.14 | −0.28(−0.37–−0.2) |
| Norway | 4.65(3.8–5.61) | 64.82(53.53–77.48) | 12.1(9.84–14.5) | 122.01(100.22–145.29) | 160.15 | 2.63(2.43–2.83) |
| Oman | 0.43(0.36–0.52) | 87.83(71.92–105.24) | 1.36(1.13–1.66) | 112.45(92.89–135.24) | 213.27 | 0.96(0.85–1.07) |
| Pakistan | 32.15(26.45–38.16) | 61.2(49.92–72.88) | 64.4(53.59–77.11) | 69.38(56.9–82.75) | 100.3 | 0.41(0.33–0.49) |
| Palau | 0.01(0.01–0.02) | 160.12(130.79–193.28) | 0.03(0.02–0.03) | 156.32(127.35–187.35) | 98.7 | −0.01(−0.08–0.06) |
| Palestine | 0.59(0.48–0.7) | 76.7(62.82–91.2) | 1.52(1.26–1.79) | 78.28(65.19–92.38) | 158.42 | 0.16(0.09–0.24) |
| Panama | 1.16(0.96–1.37) | 81.67(67.69–97.12) | 4.23(3.55–4.98) | 102.25(85.88–120.79) | 265.17 | 0.61(0.54–0.68) |
| Papua New Guinea | 1.55(1.26–1.9) | 104.27(84.2–127.09) | 3.86(3.16–4.64) | 100.36(80.9–119.88) | 148.13 | −0.05(−0.09–−0.01) |
| Paraguay | 1.52(1.26–1.77) | 72.85(60.86–84.51) | 4.61(3.87–5.39) | 87.72(74.01–102.62) | 203.92 | 0.62(0.54–0.7) |
| Peru | 9.18(7.57–10.8) | 82.33(67.96–96.92) | 32.46(27.75–37.5) | 102.04(87.14–117.64) | 253.51 | 0.78(0.69–0.86) |
| Philippines | 21.36(17.82–25.41) | 84.94(69.91–100.84) | 63.89(52.91–76.11) | 94.03(77.43–112.21) | 199.09 | 0.39(0.32–0.46) |
| Poland | 46.91(38.08–56.24) | 108.41(88.41–130.06) | 78.8(68.02–90.25) | 107.53(92.95–122.98) | 67.99 | −0.09(−0.15–−0.03) |
| Portugal | 12.7(10.48–15.36) | 90.1(74.84–108.47) | 28.98(24.77–34.13) | 111.22(95.17–130.79) | 128.2 | 0.78(0.73–0.82) |
| Puerto Rico | 3.2(2.7–3.8) | 87.82(73.98–103.88) | 7.54(6.09–9.03) | 98.58(79.98–118.55) | 135.23 | 0.16(0.08–0.23) |
| Qatar | 0.08(0.07–0.1) | 99.24(81.87–118.47) | 0.75(0.59–0.95) | 119.33(98.31–142.94) | 811.57 | 0.94(0.81–1.07) |
| Republic of Korea | 15.47(12.78–18.6) | 59.42(49.57–71.61) | 72.58(61.42–88.22) | 81.15(68.62–98.4) | 369.11 | 1.15(1.07–1.22) |
| Republic of Moldova | 3.66(2.99–4.41) | 90.63(74.59–108.81) | 4.18(3.45–4.97) | 72.04(59.57–85.35) | 14.29 | −0.99(−1.07–−0.92) |
| Romania | 19.64(16.61–24.21) | 72.65(61.25–88.84) | 33.79(28.98–40.72) | 84.67(72.31–101.26) | 71.99 | 0.58(0.54–0.62) |
| Russian Federation | 146.34(118.99–176.48) | 84.48(68.94–101.82) | 196.6(159.66–236.48) | 82.42(67.09–98.82) | 34.34 | −0.06(−0.2–0.09) |
| Rwanda | 1.33(1.1–1.65) | 53.87(44.22–65.52) | 2.85(2.35–3.47) | 56.87(46.33–69.39) | 113.87 | 0.31(0.24–0.37) |
| Saint Kitts and Nevis | 0.04(0.03–0.05) | 103.72(87.64–122.59) | 0.06(0.05–0.07) | 111.77(92.05–133.26) | 55.43 | 0.09(0–0.19) |
| Saint Lucia | 0.08(0.06–0.09) | 89.19(75.38–103.89) | 0.21(0.18–0.25) | 100.8(84.05–118.69) | 175.5 | 0.21(0.08–0.33) |
| Saint Vincent and the Grenadines | 0.06(0.04–0.07) | 77.72(62.94–93.46) | 0.11(0.09–0.14) | 87.91(72.72–104.94) | 107.88 | 0.42(0.29–0.54) |
| Samoa | 0.1(0.08–0.12) | 133.43(110.97–158.03) | 0.17(0.14–0.2) | 130.97(110.4–153.33) | 65.72 | 0.04(0–0.09) |
| San Marino | 0.03(0.03–0.04) | 101.8(87.93–119.45) | 0.07(0.06–0.09) | 105.61(88.76–124.53) | 109.07 | 0.05(0–0.11) |
| Sao Tome and Principe | 0.04(0.03–0.04) | 65.05(53.59–78.76) | 0.07(0.06–0.08) | 76.44(62.61–92.27) | 82.64 | 0.64(0.54–0.73) |
| Saudi Arabia | 3.98(3.29–4.71) | 84.28(68.75–101.1) | 13.29(11.02–15.91) | 107.61(90.36–127.75) | 233.88 | 0.86(0.8–0.91) |
| Senegal | 1.73(1.41–2.08) | 63.8(52.29–76.54) | 4.72(3.82–5.56) | 74.31(60.69–87.67) | 172.65 | 0.6(0.48–0.72) |
| Serbia | 8.52(7.1–9.92) | 82.51(68.69–95.78) | 14.76(12.19–17.48) | 86.83(72.52–102.51) | 73.18 | 0.15(0.12–0.18) |
| Seychelles | 0.07(0.06–0.08) | 119.43(100.07–139.73) | 0.13(0.11–0.15) | 131.02(107.13–158.18) | 90.96 | 0.38(0.29–0.46) |
| Sierra Leone | 1.01(0.83–1.19) | 58.27(48.54–68.62) | 2.06(1.73–2.45) | 68.04(57.29–81.19) | 103.87 | 0.58(0.49–0.68) |
| Singapore | 1.34(1.12–1.56) | 66.93(55.65–76.83) | 5.65(4.72–6.65) | 75.39(63.01–88.69) | 320.71 | 0.52(0.42–0.61) |
| Slovakia | 4.79(3.97–5.72) | 79.81(66.33–95.01) | 7.71(6.27–9.25) | 81.67(66.74–97.58) | 61.03 | 0.07(0.04–0.11) |
| Slovenia | 2.27(1.89–2.66) | 92.36(77.02–108.58) | 4.27(3.62–5.03) | 92.53(78.88–109.67) | 88.45 | −0.09(−0.15–−0.03) |
| Solomon Islands | 0.14(0.11–0.17) | 129.11(106.87–159.27) | 0.31(0.25–0.38) | 127.02(102.49–153.34) | 126.04 | −0.01(−0.06–0.03) |
| Somalia | 1.02(0.81–1.26) | 50.24(40.37–61.66) | 2.68(2.18–3.33) | 49.51(40.08–60.41) | 163.21 | 0.03(−0.02–0.08) |
| South Africa | 10.45(8.67–12.44) | 55.61(45.69–66.28) | 26.16(21.7–31.13) | 65.58(53.81–77.73) | 150.32 | 0.54(0.46–0.62) |
| South Sudan | 1.17(0.94–1.42) | 55.38(44.72–67.4) | 1.79(1.44–2.19) | 55.44(44.75–68.36) | 53.24 | 0(−0.03–0.03) |
| Spain | 67.27(58.2–76.78) | 119.15(103.5–136.13) | 129.87(104.44–156.02) | 124.73(100.32–149.01) | 93.05 | 0.04(−0.06–0.13) |
| Sri Lanka | 9.54(7.78–11.57) | 102.51(83.82–124.21) | 28.12(22.87–34.04) | 117.36(95.77–141.13) | 194.79 | 0.44(0.36–0.53) |
| Sudan | 5.63(4.58–6.69) | 68.21(55.97–80.93) | 11.4(9.53–13.65) | 69.67(58.26–83.4) | 102.42 | 0.08(0.02–0.13) |
| Suriname | 0.16(0.14–0.2) | 67.56(55.49–82.19) | 0.46(0.39–0.54) | 81.22(69.33–94.58) | 180.33 | 0.56(0.42–0.7) |
| Sweden | 17.62(14.19–21.23) | 108.44(88.77–129.35) | 28.24(22.84–34.58) | 125.21(102.54–150.36) | 60.25 | 0.6(0.56–0.64) |
| Switzerland | 12.82(10.85–14.96) | 115.13(97.26–135.31) | 22.04(18.89–25.8) | 119.15(102.4–139.46) | 71.91 | 0.37(0.28–0.47) |
| Syrian Arab Republic | 3.21(2.69–3.81) | 73.11(61.19–85.79) | 8.35(6.93–9.93) | 81.6(67.79–97.05) | 160.45 | 0.54(0.43–0.65) |
| Taiwan  (Province of China) | 18.47(15.74–21.98) | 132.94(114.07–157) | 60.08(50.45–68.01) | 151.08(126.75–171.44) | 225.38 | 1.08(0.63–1.54) |
| Tajikistan | 2.06(1.65–2.5) | 79.49(63.8–97.24) | 3.51(2.83–4.27) | 102.22(82.93–123.95) | 70.56 | 0.87(0.81–0.93) |
| Thailand | 31.36(25.68–37.47) | 105.25(86.62–126.33) | 102.6(84.98–122.79) | 103.22(85.65–123.43) | 227.17 | 0.04(−0.06–0.15) |
| Timor–Leste | 0.17(0.14–0.21) | 79.2(64.1–95.66) | 0.65(0.53–0.77) | 89.52(73.25–105.9) | 283.33 | 0.52(0.45–0.59) |
| Togo | 0.63(0.52–0.75) | 63.91(53.06–75.32) | 1.97(1.62–2.39) | 70.41(57.94–84.45) | 214.97 | 0.34(0.23–0.45) |
| Tokelau | 0(0–0) | 126.21(102.07–151.08) | 0(0–0) | 133.13(107.64–158.79) | −3.32 | 0.3(0.25–0.35) |
| Tonga | 0.06(0.05–0.07) | 123.91(102.61–148.31) | 0.09(0.07–0.11) | 117.87(96.56–139.05) | 48.74 | −0.02(−0.07–0.04) |
| Trinidad and Tobago | 0.61(0.52–0.72) | 76.42(64.58–89.19) | 1.55(1.3–1.88) | 86.05(72.47–104.25) | 151.69 | 0.25(0.17–0.33) |
| Tunisia | 3.02(2.51–3.6) | 69.18(57.3–82.09) | 9.31(7.83–10.88) | 79.64(66.89–93.19) | 208.46 | 0.52(0.49–0.54) |
| Turkey | 22.3(18.13–26.77) | 71.05(57.54–85.64) | 72.46(59.36–86.77) | 86.88(70.98–104.07) | 224.96 | 0.9(0.82–0.99) |
| Turkmenistan | 1.09(0.9–1.31) | 65.52(54.17–78) | 2.41(2–2.88) | 73.03(60.15–87.14) | 120.6 | 0.29(0.25–0.32) |
| Tuvalu | 0.01(0.01–0.01) | 118.88(97.03–141.78) | 0.01(0.01–0.01) | 128.6(105.55–153.83) | 71 | 0.36(0.3–0.42) |
| Uganda | 3.05(2.46–3.76) | 54.45(43.64–66.56) | 6.76(5.55–8.16) | 56.5(46.3–67.87) | 121.47 | 0.17(0.12–0.21) |
| Ukraine | 64.32(52.35–77.88) | 90.48(73.93–108.93) | 71.88(58.4–87.2) | 90.91(74.28–110.04) | 11.75 | −0.02(−0.07–0.03) |
| United Arab Emirates | 0.31(0.25–0.38) | 103.76(85.18–124.49) | 3.13(2.51–3.88) | 115.98(97.63–138.78) | 901.04 | 0.47(0.41–0.52) |
| United Kingdom | 129.69(108.67–153.35) | 135.58(113.99–159.39) | 168.78(139.62–199.04) | 126.86(106.53–148.11) | 30.14 | −0.43(−0.59–−0.26) |
| United Republic of Tanzania | 4.93(3.98–5.96) | 52.79(43.26–63.05) | 11.97(10.24–14.15) | 55.79(48.28–64.53) | 142.71 | 0.24(0.19–0.29) |
| United States of America | 314.24(257.01–374.83) | 93.69(77.23–111.34) | 618.19(557.33–679.01) | 106.1(95.63–116.43) | 96.72 | 0.42(0.29–0.55) |
| United States Virgin Islands | 0.08(0.07–0.09) | 107.3(90.48–125.28) | 0.22(0.18–0.27) | 120.93(100.31–146.72) | 177.23 | 0.4(0.29–0.52) |
| Uruguay | 3.43(2.92–4.19) | 85.61(72.76–104.57) | 5.12(4.29–6.29) | 88.13(73.7–108.74) | 49.38 | 0.36(0.26–0.45) |
| Uzbekistan | 7.97(6.51–9.53) | 78.04(63.57–93.59) | 13.51(11.19–16.31) | 92.44(77.27–109.45) | 69.65 | 0.52(0.47–0.58) |
| Vanuatu | 0.07(0.06–0.09) | 135.98(110.7–164.31) | 0.2(0.16–0.24) | 130.34(107.31–156.31) | 164.47 | 0(−0.06–0.06) |
| Venezuela | 6.39(5.24–7.58) | 72.27(59.12–85.04) | 24.16(20.75–28.56) | 88.03(75.47–103.18) | 278.19 | 0.58(0.51–0.65) |
| Viet Nam | 33.37(27.79–40.23) | 90.22(75.46–107.36) | 89.47(74.45–109.14) | 108.53(90.46–131.89) | 168.13 | 0.76(0.68–0.84) |
| Yemen | 2.36(1.91–2.85) | 57.97(47–69.58) | 7.21(5.87–8.68) | 63.6(51.85–76.43) | 205.09 | 0.54(0.47–0.62) |
| Zambia | 1.41(1.14–1.76) | 58.84(47.38–71.91) | 3.42(2.83–4.08) | 61.46(50.78–72.45) | 142.17 | 0.13(0.07–0.19) |
| Zimbabwe | 2.1(1.72–2.52) | 63.22(52.85–75.01) | 3.56(2.97–4.22) | 63.99(53.96–74.37) | 69.5 | −0.15(−0.26–−0.03) |

EAPC: estimated annual percentage change; ASR, age-standardized rate; CI, confidence interval; UI: uncertainty interval.

**Supplementary table 4**. The percentage changes and EAPCs of YLDs caused by Parkinson’s disease at the national level from 1990 to 2019.

|  | **1990** | | **2019** | | **1990-2019** | |
| --- | --- | --- | --- | --- | --- | --- |
| **Characteristics** | Number  ×10^3^ (95% UI) | ASR/100,000  (95% UI) | Number  ×10^3^ (95% UI) | ASR/100,000  (95% UI) | Percentage change (%) | EAPC  (95%CI) |
| Afghanistan | 0.65(0.43–0.92) | 10.04(6.78–14.03) | 1.06(0.72–1.49) | 9.63(6.49–13.27) | 63.79 | −0.08(−0.15–−0.02) |
| Albania | 0.24(0.16–0.33) | 12.89(8.73–17.49) | 0.62(0.42–0.85) | 14.05(9.47–19.16) | 160 | 0.28(0.25–0.31) |
| Algeria | 1.1(0.74–1.56) | 10.41(7.12–14.34) | 3.44(2.39–4.72) | 11.38(7.88–15.54) | 213.32 | 0.34(0.3–0.38) |
| American Samoa | 0(0–0.01) | 20.22(13.85–27.66) | 0.01(0.01–0.01) | 19.09(13.09–26.13) | 118.36 | −0.04(−0.12–0.03) |
| Andorra | 0.01(0.01–0.01) | 15.34(10.46–20.99) | 0.02(0.02–0.03) | 16.42(11.22–22.41) | 190.91 | 0.24(0.21–0.28) |
| Angola | 0.25(0.16–0.35) | 7.66(5.24–10.69) | 0.77(0.52–1.11) | 8.45(5.76–11.86) | 214.84 | 0.45(0.4–0.49) |
| Antigua and Barbuda | 0.01(0–0.01) | 12.56(8.63–17.27) | 0.01(0.01–0.02) | 14.89(10.08–20.36) | 99.26 | 0.51(0.37–0.65) |
| Argentina | 4.06(2.79–5.59) | 12.96(8.95–17.9) | 6.85(4.64–9.41) | 12.37(8.38–17.01) | 68.67 | −0.01(−0.12–0.11) |
| Armenia | 0.25(0.17–0.35) | 10.76(7.29–14.68) | 0.47(0.32–0.64) | 11.49(7.88–15.6) | 86.03 | 0.21(0.14–0.29) |
| Australia | 3.74(2.58–5.11) | 18.96(13.11–25.98) | 8.44(5.71–11.83) | 19.52(13.12–27.33) | 125.91 | −0.19(−0.5–0.11) |
| Austria | 1.91(1.3–2.59) | 15.26(10.44–20.75) | 3.47(2.42–4.81) | 18.11(12.54–25.19) | 81.56 | 0.46(0.38–0.53) |
| Azerbaijan | 0.47(0.32–0.65) | 10.77(7.36–14.87) | 0.94(0.65–1.32) | 12.69(8.91–17.28) | 99.71 | 0.47(0.42–0.51) |
| Bahamas | 0.02(0.01–0.03) | 12.69(8.67–17.49) | 0.05(0.03–0.07) | 13.84(9.65–18.94) | 171.92 | 0.16(0.07–0.25) |
| Bahrain | 0.02(0.01–0.02) | 11.66(7.92–16.04) | 0.1(0.06–0.14) | 12.97(8.8–17.7) | 494.35 | 0.44(0.39–0.49) |
| Bangladesh | 3.91(2.64–5.45) | 9.31(6.32–12.87) | 11.72(8.08–16.39) | 9.52(6.59–13.21) | 200.09 | −0.05(−0.09–0) |
| Barbados | 0.04(0.03–0.05) | 11.89(8.16–16.34) | 0.07(0.05–0.1) | 14.37(9.85–20.18) | 90.9 | 0.49(0.39–0.59) |
| Belarus | 1.49(1.02–2.08) | 11.66(8.01–16.47) | 1.95(1.34–2.77) | 12.09(8.3–17.19) | 31.35 | −0.01(−0.06–0.04) |
| Belgium | 2.48(1.74–3.4) | 15.46(10.82–21.12) | 4.17(2.83–5.74) | 17.23(11.66–23.97) | 67.97 | 0.62(0.56–0.68) |
| Belize | 0.01(0.01–0.01) | 11.74(8.03–16.36) | 0.03(0.02–0.05) | 13.27(9.13–18) | 214.34 | 0.31(0.18–0.44) |
| Benin | 0.17(0.11–0.23) | 9.2(6.22–12.73) | 0.42(0.28–0.6) | 10.24(6.97–14.18) | 154.19 | 0.37(0.24–0.49) |
| Bermuda | 0.01(0.01–0.01) | 15.66(10.81–21.39) | 0.02(0.02–0.03) | 16.47(11.31–22.52) | 138.77 | 0.13(0.01–0.26) |
| Bhutan | 0.02(0.01–0.03) | 8.72(5.84–12.13) | 0.05(0.04–0.08) | 10.43(7.06–14.43) | 200.81 | 0.56(0.52–0.6) |
| Bolivia | 0.36(0.24–0.5) | 11.98(8.18–16.59) | 1.1(0.76–1.52) | 13.18(9.18–18.09) | 203.66 | 0.24(0.17–0.31) |
| Bosnia and Herzegovina | 0.47(0.31–0.65) | 13.03(8.8–18.01) | 0.79(0.54–1.13) | 12.99(8.91–18.35) | 70.12 | 0.03(0–0.06) |
| Botswana | 0.04(0.03–0.05) | 7.68(5.28–10.58) | 0.11(0.08–0.15) | 9.83(6.85–13.35) | 197.04 | 0.78(0.71–0.85) |
| Brazil | 8.39(5.7–11.66) | 10.35(7.02–14.13) | 28.67(19.51–39.39) | 12.46(8.47–17.12) | 241.82 | 0.65(0.55–0.74) |
| Brunei Darussalam | 0.01(0.01–0.01) | 10.8(7.36–14.95) | 0.03(0.02–0.05) | 12.91(8.74–17.79) | 259.71 | 0.73(0.64–0.82) |
| Bulgaria | 1.69(1.15–2.33) | 14.05(9.59–19.15) | 2.06(1.37–2.82) | 12.98(8.68–17.68) | 21.29 | −0.61(−0.74–−0.49) |
| Burkina Faso | 0.32(0.21–0.45) | 8.75(5.84–12.18) | 0.75(0.5–1.05) | 9.87(6.73–13.89) | 133.69 | 0.48(0.36–0.6) |
| Burundi | 0.16(0.11–0.23) | 7.65(5.15–10.58) | 0.31(0.21–0.44) | 8.05(5.35–11.16) | 90.49 | 0.25(0.19–0.31) |
| Cabo Verde | 0.02(0.01–0.03) | 8.56(5.73–11.99) | 0.04(0.03–0.06) | 10.56(7.24–14.29) | 107.92 | 0.81(0.73–0.9) |
| Cambodia | 0.45(0.3–0.63) | 11.75(7.88–16.23) | 1.34(0.92–1.84) | 12.6(8.81–17.18) | 196.56 | 0.32(0.23–0.4) |
| Cameroon | 0.36(0.25–0.5) | 9.86(6.81–13.58) | 1.1(0.76–1.53) | 11.42(8.01–15.75) | 208.59 | 0.58(0.47–0.69) |
| Canada | 3.75(2.58–5.13) | 11.42(7.84–15.6) | 12.26(8.45–17.15) | 17.09(11.81–23.81) | 226.82 | 2.13(1.47–2.79) |
| Central African Republic | 0.07(0.05–0.1) | 7.49(5.07–10.81) | 0.14(0.09–0.19) | 7.57(5.02–10.66) | 86.67 | 0.13(0.09–0.16) |
| Chad | 0.22(0.15–0.3) | 8.53(5.79–11.72) | 0.45(0.31–0.62) | 9.59(6.41–13.22) | 106.31 | 0.42(0.34–0.5) |
| Chile | 1.12(0.78–1.53) | 12.06(8.47–16.48) | 3.18(2.17–4.44) | 13.21(8.99–18.41) | 185.35 | 0.63(0.53–0.73) |
| China | 114.89(76.81–160.95) | 15.31(10.36–21.17) | 411.66(276.75–571.46) | 20.9(14.1–28.98) | 258.32 | 1.03(0.75–1.3) |
| Colombia | 1.68(1.14–2.35) | 10.53(7.17–14.52) | 6.55(4.52–9.08) | 12.31(8.44–17.13) | 290.44 | 0.48(0.39–0.57) |
| Comoros | 0.02(0.01–0.02) | 8.17(5.52–11.29) | 0.04(0.03–0.05) | 8.64(5.97–12.13) | 133.44 | 0.21(0.19–0.23) |
| Congo | 0.08(0.05–0.11) | 8.32(5.58–11.58) | 0.2(0.14–0.28) | 9.11(6.22–12.44) | 163.45 | 0.43(0.38–0.48) |
| Cook Islands | 0(0–0) | 21.33(14.64–28.95) | 0(0–0.01) | 19.99(13.61–27.69) | 104.83 | −0.07(−0.13–−0.01) |
| Costa Rica | 0.2(0.14–0.28) | 12.21(8.23–16.9) | 0.7(0.48–0.95) | 13.84(9.54–18.93) | 242.85 | 0.4(0.36–0.45) |
| Croatia | 0.76(0.52–1.05) | 12.21(8.42–16.79) | 1.28(0.88–1.77) | 13.51(9.25–18.61) | 69.15 | 0.25(0.2–0.3) |
| Cuba | 1.08(0.74–1.49) | 10.56(7.3–14.48) | 2.59(1.77–3.62) | 13.31(9.12–18.59) | 138.77 | 0.91(0.8–1.03) |
| Cyprus | 0.14(0.09–0.2) | 17.61(11.62–24.5) | 0.35(0.24–0.49) | 17.89(12.19–24.59) | 147.09 | 0.1(0.08–0.12) |
| Czechia | 1.59(1.08–2.23) | 11.27(7.65–15.64) | 2.76(1.86–3.75) | 12.34(8.34–16.85) | 73.37 | 0.21(0.14–0.29) |
| Côte d'Ivoire | 0.3(0.21–0.43) | 10.08(6.99–13.94) | 0.93(0.63–1.33) | 11.17(7.64–15.62) | 206.82 | 0.39(0.29–0.48) |
| Democratic People's Republic of Korea | 2.24(1.52–3.11) | 16.19(11.08–22.52) | 4.57(3.09–6.4) | 14.68(10.06–20.51) | 103.9 | −0.33(−0.4–−0.27) |
| Democratic Republic of the Congo | 1.01(0.67–1.43) | 7.78(5.34–10.87) | 2.49(1.65–3.54) | 8.18(5.51–11.59) | 146.69 | 0.19(0.11–0.27) |
| Denmark | 1.1(0.75–1.5) | 12.85(8.94–17.63) | 1.99(1.39–2.7) | 16.5(11.57–22.61) | 81.26 | 1.14(1.06–1.23) |
| Djibouti | 0.01(0.01–0.01) | 7.9(5.4–11.04) | 0.04(0.03–0.06) | 8.74(5.89–12.14) | 378.46 | 0.4(0.35–0.44) |
| Dominica | 0.01(0.01–0.01) | 12.61(8.52–17.44) | 0.01(0.01–0.02) | 13.5(9.31–18.66) | 29.37 | 0.13(−0.02–0.28) |
| Dominican Republic | 0.34(0.24–0.48) | 9.9(6.84–13.73) | 1.13(0.76–1.56) | 12.69(8.57–17.54) | 230.06 | 0.7(0.61–0.79) |
| Ecuador | 0.54(0.37–0.74) | 10.59(7.27–14.56) | 2.12(1.44–2.9) | 14.59(9.94–19.89) | 295.48 | 1.06(0.96–1.16) |
| Egypt | 2.57(1.69–3.62) | 10.29(6.94–14.33) | 6.37(4.28–8.93) | 11.99(8.21–16.51) | 147.55 | 0.49(0.46–0.52) |
| El Salvador | 0.3(0.2–0.4) | 10.48(7.17–14.22) | 0.79(0.54–1.08) | 12.94(8.89–17.7) | 165.89 | 0.67(0.57–0.77) |
| Equatorial Guinea | 0.01(0.01–0.02) | 7.36(4.93–10.09) | 0.04(0.03–0.06) | 9.5(6.3–13.31) | 214.27 | 1.07(1.01–1.14) |
| Eritrea | 0.06(0.04–0.08) | 7.18(4.75–10.05) | 0.19(0.12–0.27) | 8.31(5.64–11.51) | 219.53 | 0.46(0.44–0.48) |
| Estonia | 0.28(0.2–0.39) | 13.76(9.59–19.04) | 0.37(0.25–0.51) | 13.11(8.75–18.07) | 30.23 | −0.27(−0.34–−0.2) |
| Eswatini | 0.02(0.01–0.03) | 8(5.45–11.01) | 0.04(0.03–0.06) | 8.95(6.23–12.22) | 119.29 | 0.24(0.16–0.33) |
| Ethiopia | 1.31(0.88–1.83) | 7.64(5.16–10.56) | 2.99(2–4.16) | 7.98(5.39–11.03) | 128.09 | 0.15(0.11–0.19) |
| Fiji | 0.06(0.04–0.08) | 19.71(13.43–27.8) | 0.12(0.08–0.16) | 18.03(12.37–25.1) | 102.34 | −0.16(−0.22–−0.1) |
| Finland | 1.08(0.75–1.45) | 14.68(10.22–19.98) | 2.38(1.65–3.34) | 18.3(12.65–25.67) | 121.1 | 0.72(0.67–0.77) |
| France | 12.96(8.89–17.52) | 14.78(10.08–20.1) | 25.26(17.6–34.31) | 17.3(11.93–23.76) | 94.98 | 0.72(0.62–0.82) |
| Gabon | 0.05(0.03–0.07) | 9.4(6.34–13.07) | 0.09(0.06–0.13) | 10.38(7.05–14) | 99.67 | 0.41(0.36–0.45) |
| Gambia | 0.03(0.02–0.04) | 9.2(6.4–12.54) | 0.09(0.06–0.13) | 10.75(7.44–14.67) | 238.51 | 0.5(0.41–0.6) |
| Georgia | 0.68(0.46–0.96) | 11.7(7.97–16.44) | 0.66(0.45–0.91) | 10.64(7.24–14.64) | −3.39 | −0.53(−0.6–−0.46) |
| Germany | 19.09(13.07–26.27) | 14.5(9.91–19.94) | 38.21(26.17–52.13) | 18.49(12.63–25.32) | 100.13 | 1.64(1.3–1.99) |
| Ghana | 0.44(0.29–0.62) | 8.77(6.01–12.1) | 1.33(0.89–1.89) | 10.01(6.75–13.9) | 203.33 | 0.45(0.32–0.58) |
| Greece | 2.38(1.61–3.27) | 15.29(10.3–20.95) | 4.28(3.03–5.86) | 16.58(11.67–22.55) | 80.14 | 0.3(0.26–0.34) |
| Greenland | 0(0–0.01) | 13.94(9.55–19.34) | 0.01(0.01–0.01) | 15.38(10.49–21.41) | 148.13 | 0.37(0.35–0.4) |
| Grenada | 0.01(0.01–0.01) | 10.94(7.48–14.9) | 0.01(0.01–0.02) | 13.55(9.28–18.71) | 68.22 | 0.58(0.5–0.66) |
| Guam | 0.01(0.01–0.02) | 21.09(14.35–28.89) | 0.03(0.02–0.04) | 17.75(12.37–24.02) | 150.66 | −0.6(−0.67–−0.53) |
| Guatemala | 0.32(0.21–0.45) | 10.19(6.94–14.17) | 1.21(0.83–1.66) | 11.22(7.74–15.52) | 277.43 | 0.18(0.09–0.27) |
| Guinea | 0.26(0.18–0.37) | 9.02(6.17–12.61) | 0.51(0.35–0.71) | 10.57(7.22–14.48) | 92.3 | 0.55(0.43–0.67) |
| Guinea–Bissau | 0.03(0.02–0.04) | 9.31(6.35–12.86) | 0.06(0.04–0.08) | 10(6.74–13.91) | 84.88 | 0.26(0.17–0.36) |
| Guyana | 0.04(0.03–0.06) | 11.29(7.72–15.86) | 0.07(0.05–0.1) | 12.53(8.55–17.14) | 79.8 | 0.28(0.14–0.41) |
| Haiti | 0.28(0.19–0.4) | 9.77(6.41–13.65) | 0.7(0.47–0.98) | 11.57(7.85–15.93) | 151.16 | 0.5(0.41–0.6) |
| Honduras | 0.2(0.14–0.29) | 10.76(7.24–14.86) | 0.7(0.48–0.96) | 12.48(8.57–17.02) | 241.92 | 0.42(0.36–0.47) |
| Hungary | 1.63(1.12–2.2) | 10.78(7.44–14.54) | 2.43(1.66–3.31) | 11.83(8.15–16.25) | 49.45 | 0.38(0.28–0.47) |
| Iceland | 0.05(0.04–0.07) | 18.15(12.27–24.44) | 0.12(0.08–0.16) | 21.04(13.99–28.47) | 124.5 | 0.56(0.51–0.61) |
| India | 32.69(22.02–45.56) | 8.53(5.79–11.72) | 107.98(72.77–149.48) | 10.22(6.9–14.04) | 230.31 | 0.62(0.58–0.65) |
| Indonesia | 9.75(6.61–13.56) | 11.73(7.9–16.01) | 23.73(15.88–32.75) | 12.59(8.43–17.34) | 143.39 | 0.22(0.15–0.29) |
| Iran  (Islamic Republic of) | 2.27(1.53–3.19) | 10.61(7.14–14.57) | 8.07(5.47–11.1) | 12(8.09–16.59) | 255.11 | 0.39(0.33–0.45) |
| Iraq | 0.69(0.47–0.96) | 9.8(6.7–13.58) | 2.07(1.43–2.83) | 10.47(7.26–14.17) | 199.97 | 0.25(0.2–0.29) |
| Ireland | 0.67(0.45–0.91) | 15.83(10.8–21.62) | 1.44(0.99–2) | 18.74(12.89–26.1) | 115.95 | 0.65(0.59–0.7) |
| Israel | 0.83(0.57–1.14) | 16.67(11.57–22.92) | 1.97(1.35–2.75) | 16.59(11.32–23.23) | 138.66 | 0.06(−0.02–0.15) |
| Italy | 21.55(14.76–29.51) | 23.18(15.93–31.6) | 28.84(19.62–39.47) | 18.59(12.58–25.54) | 33.86 | −1.01(−1.3–−0.72) |
| Jamaica | 0.2(0.14–0.28) | 11.11(7.55–15.53) | 0.38(0.26–0.52) | 12.73(8.8–17.18) | 88.6 | 0.47(0.3–0.64) |
| Japan | 15.07(10.16–20.82) | 8.93(6.05–12.32) | 42.55(28.76–58.81) | 11.21(7.54–15.48) | 182.4 | 0.46(0.33–0.59) |
| Jordan | 0.11(0.08–0.16) | 10.62(7.32–14.36) | 0.6(0.41–0.84) | 10.92(7.52–15.11) | 425.01 | −0.05(−0.18–0.07) |
| Kazakhstan | 1.24(0.84–1.73) | 10.85(7.44–14.97) | 1.84(1.25–2.58) | 11.95(8.18–16.67) | 48.46 | 0.31(0.21–0.42) |
| Kenya | 0.56(0.38–0.78) | 7.68(5.15–10.57) | 1.55(1.04–2.15) | 8.04(5.4–11.12) | 175.24 | 0.17(0.14–0.21) |
| Kiribati | 0.01(0–0.01) | 16.8(11.46–22.91) | 0.01(0.01–0.01) | 15.85(10.74–21.92) | 70.56 | −0.12(−0.16–−0.08) |
| Kuwait | 0.06(0.04–0.08) | 11.14(7.58–15.39) | 0.23(0.16–0.33) | 10.11(6.83–13.87) | 311.65 | −0.33(−0.4–−0.26) |
| Kyrgyzstan | 0.28(0.19–0.39) | 9.81(6.54–13.68) | 0.38(0.25–0.52) | 9.38(6.35–12.87) | 33.28 | −0.3(−0.34–−0.26) |
| Lao People's Democratic Republic | 0.21(0.14–0.29) | 11.61(7.89–15.93) | 0.48(0.33–0.66) | 12.73(8.84–17.32) | 130.99 | 0.28(0.2–0.36) |
| Latvia | 0.45(0.31–0.62) | 12.47(8.54–16.97) | 0.52(0.35–0.72) | 12.17(8.23–16.85) | 15.53 | −0.16(−0.24–−0.09) |
| Lebanon | 0.2(0.14–0.28) | 10.07(6.84–13.82) | 0.57(0.4–0.78) | 10.85(7.53–14.94) | 183.98 | 0.37(0.32–0.41) |
| Lesotho | 0.06(0.04–0.09) | 7.18(4.97–9.86) | 0.08(0.06–0.12) | 7.83(5.41–10.76) | 34.66 | 0.11(0.02–0.2) |
| Liberia | 0.09(0.06–0.12) | 9.09(6.14–12.44) | 0.17(0.11–0.24) | 9.9(6.75–13.74) | 87.36 | 0.32(0.18–0.46) |
| Libya | 0.18(0.12–0.24) | 10.57(7.3–14.39) | 0.55(0.37–0.76) | 11.84(8.05–16.26) | 210.77 | 0.56(0.49–0.64) |
| Lithuania | 0.52(0.35–0.72) | 11.47(7.76–15.8) | 0.75(0.51–1.03) | 12.17(8.28–16.77) | 44.43 | 0.27(0.21–0.32) |
| Luxembourg | 0.09(0.06–0.13) | 16.74(11.34–22.98) | 0.2(0.13–0.27) | 19.15(12.98–26.55) | 108.32 | 0.42(0.32–0.53) |
| Madagascar | 0.32(0.21–0.46) | 7.11(4.7–9.92) | 0.69(0.45–0.99) | 7.54(5.07–10.64) | 113.27 | 0.19(0.15–0.23) |
| Malawi | 0.26(0.17–0.36) | 7.86(5.26–10.82) | 0.52(0.34–0.73) | 8.11(5.44–11.26) | 100.37 | 0.16(0.13–0.19) |
| Malaysia | 1.11(0.75–1.55) | 13.69(9.2–18.98) | 3.71(2.48–5.22) | 15.26(10.32–21.33) | 234.41 | 0.41(0.33–0.48) |
| Maldives | 0.01(0.01–0.01) | 15.03(10.34–20.69) | 0.04(0.03–0.06) | 16.06(10.98–22) | 316.56 | 0.32(0.22–0.42) |
| Mali | 0.29(0.2–0.42) | 8.52(5.82–11.99) | 0.7(0.48–0.98) | 9.81(6.75–13.4) | 142.13 | 0.48(0.32–0.63) |
| Malta | 0.07(0.05–0.09) | 15.62(10.83–21.68) | 0.18(0.12–0.25) | 18.28(12.55–25.24) | 169.94 | 0.63(0.57–0.69) |
| Marshall Islands | 0(0–0) | 20.39(14.04–28.43) | 0.01(0–0.01) | 20.6(14.09–28.35) | 92.04 | 0.18(0.13–0.23) |
| Mauritania | 0.08(0.06–0.11) | 9.14(6.27–12.86) | 0.18(0.12–0.25) | 9.88(6.75–13.77) | 123.69 | 0.16(0.03–0.3) |
| Mauritius | 0.1(0.07–0.13) | 14.67(10.04–20.73) | 0.25(0.17–0.35) | 15.11(10.32–21.13) | 162.27 | 0.02(−0.06–0.1) |
| Mexico | 4.24(2.87–5.81) | 10.76(7.29–14.67) | 14.68(9.87–20.12) | 13.11(8.83–17.9) | 246.29 | 0.58(0.48–0.69) |
| Micronesia  (Federated States of) | 0.01(0.01–0.01) | 18.93(12.94–26.13) | 0.01(0.01–0.02) | 19.63(13.37–26.93) | 47.2 | 0.21(0.17–0.25) |
| Monaco | 0.01(0.01–0.02) | 17.21(11.53–23.65) | 0.02(0.01–0.03) | 20.44(13.99–28.14) | 56.94 | 0.66(0.63–0.68) |
| Mongolia | 0.1(0.07–0.14) | 10.57(7.11–14.42) | 0.18(0.12–0.26) | 9.6(6.56–13.32) | 79.76 | −0.39(−0.48–−0.3) |
| Montenegro | 0.08(0.05–0.11) | 13.05(8.71–18.15) | 0.14(0.09–0.2) | 13.93(9.43–19.6) | 81.71 | 0.3(0.2–0.39) |
| Morocco | 1.07(0.71–1.53) | 8.79(5.86–12.3) | 2.96(2.03–4.16) | 10.49(7.2–14.62) | 176.25 | 0.72(0.67–0.78) |
| Mozambique | 0.38(0.25–0.53) | 7.34(4.96–10.16) | 0.76(0.51–1.1) | 8.01(5.4–11.26) | 100.63 | 0.31(0.28–0.34) |
| Myanmar | 2.5(1.68–3.55) | 12.44(8.69–17.33) | 5.96(4.09–8.18) | 14.32(9.82–19.56) | 137.87 | 0.55(0.49–0.61) |
| Namibia | 0.05(0.04–0.07) | 8.15(5.6–11.18) | 0.12(0.08–0.17) | 9.61(6.67–13.27) | 130.43 | 0.51(0.41–0.6) |
| Nauru | 0(0–0) | 20.39(13.89–28.35) | 0(0–0) | 19.51(13.31–27.21) | −1.72 | 0.05(−0.03–0.13) |
| Nepal | 0.6(0.4–0.86) | 7.45(5.08–10.47) | 1.85(1.24–2.56) | 9.06(6.12–12.55) | 207.91 | 0.68(0.63–0.73) |
| Netherlands | 3.92(2.77–5.24) | 18.85(13.33–25.17) | 6.51(4.48–8.79) | 18.25(12.64–24.65) | 66.14 | −0.33(−0.48–−0.17) |
| New Zealand | 0.61(0.41–0.86) | 15.42(10.33–21.41) | 1.43(0.96–1.99) | 17.85(12.06–24.59) | 133.12 | 0.61(0.52–0.69) |
| Nicaragua | 0.14(0.09–0.19) | 9.7(6.49–13.47) | 0.47(0.32–0.67) | 11.54(7.94–16.04) | 243.23 | 0.59(0.54–0.63) |
| Niger | 0.19(0.13–0.27) | 8.68(5.98–11.95) | 0.59(0.39–0.83) | 9.49(6.4–13) | 205.99 | 0.35(0.23–0.47) |
| Nigeria | 3.28(2.22–4.56) | 8.72(5.91–11.96) | 7.22(4.89–9.98) | 10.08(6.85–13.85) | 120.35 | 0.5(0.39–0.61) |
| Niue | 0(0–0) | 19.98(13.64–27.72) | 0(0–0) | 20.41(14.16–27.95) | −4.83 | 0.21(0.15–0.27) |
| North Macedonia | 0.21(0.14–0.29) | 12.37(8.61–16.99) | 0.43(0.29–0.6) | 13.71(9.51–18.92) | 105.2 | 0.41(0.36–0.46) |
| Northern Mariana Islands | 0(0–0) | 24.56(16.75–33.7) | 0.01(0.01–0.01) | 22.2(15.23–30.43) | 203.39 | −0.32(−0.4–−0.24) |
| Norway | 0.65(0.44–0.91) | 9.22(6.29–12.66) | 1.69(1.14–2.34) | 17.24(11.72–23.88) | 159.12 | 2.61(2.41–2.8) |
| Oman | 0.06(0.04–0.09) | 12.32(8.6–16.58) | 0.2(0.13–0.28) | 15.61(10.63–21.45) | 214.04 | 0.92(0.81–1.03) |
| Pakistan | 4.59(3.06–6.38) | 8.63(5.79–12.02) | 9.25(6.26–12.85) | 9.73(6.61–13.36) | 101.75 | 0.39(0.31–0.47) |
| Palau | 0(0–0) | 22.46(15.41–31.2) | 0(0–0.01) | 21.67(14.84–30.37) | 97.72 | −0.05(−0.12–0.01) |
| Palestine | 0.08(0.06–0.12) | 10.85(7.53–14.86) | 0.22(0.15–0.31) | 10.98(7.59–15.08) | 159.17 | 0.13(0.06–0.2) |
| Panama | 0.17(0.12–0.23) | 11.7(8.13–16.1) | 0.6(0.41–0.83) | 14.56(10.01–20) | 260.45 | 0.59(0.52–0.66) |
| Papua New Guinea | 0.22(0.15–0.32) | 14.67(9.87–20.28) | 0.56(0.37–0.79) | 14.13(9.66–19.81) | 148.67 | −0.04(−0.08–0.01) |
| Paraguay | 0.22(0.15–0.3) | 10.5(7.15–14.29) | 0.66(0.45–0.91) | 12.58(8.61–17.08) | 201.59 | 0.61(0.53–0.68) |
| Peru | 1.34(0.9–1.89) | 11.92(8.09–16.72) | 4.67(3.29–6.36) | 14.66(10.32–19.98) | 247.8 | 0.76(0.68–0.85) |
| Philippines | 3.07(2.06–4.25) | 11.95(8.02–16.4) | 9.2(6.23–12.88) | 13.31(8.96–18.36) | 199.56 | 0.41(0.34–0.48) |
| Poland | 6.59(4.46–9.07) | 15.21(10.28–20.97) | 11.1(7.72–14.78) | 15.2(10.59–20.29) | 68.27 | −0.06(−0.12–0) |
| Portugal | 1.8(1.25–2.48) | 12.79(8.93–17.45) | 4.08(2.78–5.63) | 15.87(10.75–21.76) | 126.68 | 0.8(0.76–0.84) |
| Puerto Rico | 0.46(0.31–0.63) | 12.61(8.57–17.15) | 1.06(0.73–1.47) | 14.03(9.59–19.45) | 130.43 | 0.14(0.07–0.21) |
| Qatar | 0.01(0.01–0.02) | 13.88(9.34–18.83) | 0.11(0.07–0.16) | 16.38(11.3–22.51) | 807.27 | 0.87(0.75–0.99) |
| Republic of Korea | 2.26(1.53–3.12) | 8.49(5.76–11.66) | 10.45(7.17–14.49) | 11.68(7.98–16.14) | 363.12 | 1.18(1.1–1.25) |
| Republic of Moldova | 0.52(0.34–0.72) | 12.81(8.54–17.52) | 0.6(0.41–0.83) | 10.31(6.97–14.29) | 14.76 | −0.96(−1.03–−0.88) |
| Romania | 2.8(1.9–3.94) | 10.32(7.09–14.36) | 4.79(3.3–6.61) | 12.07(8.26–16.64) | 71 | 0.6(0.55–0.65) |
| Russian Federation | 20.62(13.93–28.63) | 11.86(7.97–16.39) | 27.8(18.6–38.3) | 11.68(7.86–16.14) | 34.82 | −0.02(−0.17–0.12) |
| Rwanda | 0.19(0.13–0.27) | 7.7(5.14–10.63) | 0.42(0.27–0.59) | 8.15(5.46–11.29) | 114.94 | 0.33(0.26–0.4) |
| Saint Kitts and Nevis | 0.01(0–0.01) | 14.78(10.13–20.31) | 0.01(0.01–0.01) | 15.82(11.01–21.29) | 56.79 | 0.08(−0.01–0.18) |
| Saint Lucia | 0.01(0.01–0.02) | 12.68(8.78–17.5) | 0.03(0.02–0.04) | 14.28(9.7–19.96) | 174.1 | 0.21(0.08–0.33) |
| Saint Vincent and the Grenadines | 0.01(0.01–0.01) | 11.18(7.64–15.48) | 0.02(0.01–0.02) | 12.51(8.48–17.55) | 105.97 | 0.39(0.26–0.51) |
| Samoa | 0.01(0.01–0.02) | 18.82(12.9–25.92) | 0.02(0.02–0.03) | 18.34(12.84–24.71) | 64.24 | 0.02(−0.03–0.07) |
| San Marino | 0(0–0.01) | 14.62(10.22–20.12) | 0.01(0.01–0.01) | 15.12(10.6–20.93) | 106.48 | 0.05(0–0.1) |
| Sao Tome and Principe | 0.01(0–0.01) | 9.26(6.36–13.04) | 0.01(0.01–0.01) | 10.84(7.32–15.04) | 83.25 | 0.62(0.53–0.71) |
| Saudi Arabia | 0.57(0.38–0.79) | 11.67(7.89–16.05) | 1.93(1.29–2.73) | 14.84(10.25–20.44) | 239.41 | 0.84(0.79–0.9) |
| Senegal | 0.25(0.17–0.35) | 9.04(6.19–12.56) | 0.68(0.46–0.95) | 10.55(7.13–14.63) | 172.89 | 0.59(0.48–0.7) |
| Serbia | 1.22(0.81–1.68) | 11.71(7.88–15.94) | 2.07(1.41–2.85) | 12.24(8.46–16.8) | 70.35 | 0.11(0.08–0.14) |
| Seychelles | 0.01(0.01–0.01) | 16.97(11.64–22.99) | 0.02(0.01–0.03) | 18.29(12.76–25.32) | 89.65 | 0.31(0.23–0.39) |
| Sierra Leone | 0.15(0.1–0.2) | 8.3(5.71–11.37) | 0.3(0.2–0.41) | 9.69(6.67–13.33) | 104.91 | 0.58(0.5–0.67) |
| Singapore | 0.2(0.13–0.27) | 9.65(6.65–13.17) | 0.83(0.57–1.13) | 10.98(7.61–14.94) | 321.17 | 0.56(0.47–0.66) |
| Slovakia | 0.68(0.47–0.92) | 11.27(7.76–15.4) | 1.09(0.73–1.53) | 11.57(7.78–16.2) | 61.22 | 0.08(0.04–0.12) |
| Slovenia | 0.32(0.21–0.43) | 12.94(8.62–17.74) | 0.6(0.4–0.82) | 13.03(8.78–18) | 87.6 | −0.06(−0.12–−0.01) |
| Solomon Islands | 0.02(0.01–0.03) | 18.44(12.58–25.67) | 0.05(0.03–0.06) | 18(12.22–25.13) | 124.7 | −0.05(−0.09–−0.01) |
| Somalia | 0.15(0.1–0.22) | 7.23(4.85–10.08) | 0.4(0.27–0.57) | 7.1(4.76–9.91) | 162.09 | 0.02(−0.02–0.07) |
| South Africa | 1.49(1–2.09) | 7.84(5.25–10.85) | 3.72(2.54–5.1) | 9.2(6.26–12.6) | 149.09 | 0.52(0.44–0.6) |
| South Sudan | 0.17(0.11–0.23) | 7.73(5.22–10.76) | 0.26(0.17–0.36) | 7.74(5.32–10.74) | 54.58 | 0(−0.03–0.04) |
| Spain | 9.5(6.66–12.67) | 16.86(11.84–22.53) | 18.25(12.25–25.18) | 17.75(11.93–24.51) | 92.09 | 0.07(−0.03–0.16) |
| Sri Lanka | 1.37(0.93–1.9) | 14.46(9.96–19.89) | 3.94(2.69–5.42) | 16.31(11.2–22.29) | 188.28 | 0.38(0.31–0.46) |
| Sudan | 0.82(0.55–1.13) | 9.75(6.66–13.25) | 1.65(1.11–2.32) | 9.92(6.75–14.01) | 102.26 | 0.06(0.01–0.12) |
| Suriname | 0.02(0.02–0.03) | 9.76(6.6–13.66) | 0.07(0.05–0.09) | 11.54(8.04–15.8) | 175.19 | 0.5(0.36–0.63) |
| Sweden | 2.51(1.66–3.49) | 15.57(10.35–21.6) | 3.98(2.67–5.51) | 17.85(11.93–24.83) | 58.59 | 0.58(0.54–0.62) |
| Switzerland | 1.8(1.24–2.44) | 16.26(11.27–22.18) | 3.12(2.2–4.23) | 17.04(11.85–23.26) | 73.26 | 0.4(0.3–0.49) |
| Syrian Arab Republic | 0.46(0.32–0.65) | 10.41(7.2–14.4) | 1.2(0.82–1.67) | 11.46(7.96–15.85) | 158.53 | 0.49(0.38–0.61) |
| Taiwan  (Province of China) | 2.68(1.81–3.63) | 19.04(12.97–25.74) | 8.6(5.93–11.46) | 21.65(14.87–28.82) | 220.9 | 1.07(0.62–1.53) |
| Tajikistan | 0.3(0.2–0.41) | 11.49(7.62–15.89) | 0.52(0.35–0.72) | 14.68(10.12–20.08) | 73.21 | 0.86(0.8–0.91) |
| Thailand | 4.5(3.01–6.29) | 14.84(10.08–20.53) | 14.63(9.93–20) | 14.69(10.01–20.16) | 225.21 | 0.09(−0.01–0.18) |
| Timor–Leste | 0.02(0.02–0.04) | 11.15(7.6–15.38) | 0.09(0.06–0.13) | 12.56(8.63–17.12) | 273.16 | 0.54(0.46–0.62) |
| Togo | 0.09(0.06–0.13) | 9.12(6.24–12.5) | 0.29(0.19–0.41) | 10.04(6.87–13.87) | 215.15 | 0.34(0.24–0.45) |
| Tokelau | 0(0–0) | 17.87(12.15–24.82) | 0(0–0) | 18.7(12.58–25.66) | −3.61 | 0.26(0.21–0.3) |
| Tonga | 0.01(0.01–0.01) | 17.6(12.04–24.11) | 0.01(0.01–0.02) | 16.68(11.32–22.78) | 46.77 | −0.04(−0.09–0.02) |
| Trinidad and Tobago | 0.09(0.06–0.12) | 10.98(7.71–15.22) | 0.22(0.15–0.31) | 12.26(8.5–17.16) | 148.4 | 0.23(0.15–0.31) |
| Tunisia | 0.44(0.29–0.61) | 9.87(6.76–13.59) | 1.32(0.9–1.81) | 11.24(7.7–15.22) | 202.62 | 0.48(0.46–0.5) |
| Turkey | 3.21(2.17–4.5) | 10.1(6.82–14.09) | 10.29(7.02–14.16) | 12.28(8.38–16.81) | 220.93 | 0.85(0.77–0.92) |
| Turkmenistan | 0.16(0.11–0.22) | 9.48(6.57–13.07) | 0.35(0.23–0.5) | 10.61(7.04–14.65) | 121.94 | 0.3(0.27–0.34) |
| Tuvalu | 0(0–0) | 16.85(11.36–22.92) | 0(0–0) | 18.03(12.35–24.8) | 67.99 | 0.32(0.26–0.37) |
| Uganda | 0.44(0.29–0.62) | 7.74(5.12–10.75) | 0.99(0.65–1.39) | 8.09(5.38–11.18) | 124.03 | 0.21(0.16–0.25) |
| Ukraine | 9.09(6.1–12.71) | 12.77(8.63–17.85) | 10.21(6.87–14.4) | 12.95(8.71–18.16) | 12.25 | 0.02(−0.03–0.07) |
| United Arab Emirates | 0.05(0.03–0.07) | 14.56(9.87–20.2) | 0.47(0.31–0.71) | 16.14(11.33–22.35) | 907.82 | 0.44(0.39–0.5) |
| United Kingdom | 18.35(12.63–25.01) | 19.29(13.38–26.35) | 23.64(16.32–32.18) | 17.93(12.29–24.56) | 28.79 | −0.45(−0.61–−0.28) |
| United Republic of Tanzania | 0.72(0.48–1.01) | 7.51(5.1–10.38) | 1.75(1.2–2.44) | 8.01(5.53–11.13) | 144.53 | 0.28(0.24–0.33) |
| United States of America | 43.16(29.13–59.35) | 12.94(8.75–17.74) | 83.12(59.69–107.33) | 14.35(10.26–18.56) | 92.6 | 0.32(0.19–0.46) |
| United States Virgin Islands | 0.01(0.01–0.02) | 15.49(10.41–21.34) | 0.03(0.02–0.05) | 17.24(11.75–24.16) | 170.85 | 0.36(0.25–0.48) |
| Uruguay | 0.49(0.33–0.7) | 12.3(8.21–17.29) | 0.73(0.51–1.02) | 12.62(8.71–17.67) | 47.79 | 0.34(0.25–0.43) |
| Uzbekistan | 1.16(0.78–1.59) | 11.29(7.7–15.38) | 1.99(1.31–2.82) | 13.28(8.94–18.3) | 71.9 | 0.5(0.45–0.55) |
| Vanuatu | 0.01(0.01–0.02) | 19.25(13.29–26.9) | 0.03(0.02–0.04) | 18.4(12.65–25.21) | 162.81 | −0.01(−0.07–0.05) |
| Venezuela | 0.92(0.62–1.29) | 10.32(6.95–14.27) | 3.46(2.41–4.78) | 12.55(8.7–17.11) | 274.58 | 0.58(0.5–0.65) |
| Viet Nam | 4.8(3.24–6.72) | 12.87(8.81–17.85) | 12.88(8.75–17.98) | 15.43(10.49–21.41) | 168.23 | 0.75(0.67–0.82) |
| Yemen | 0.34(0.23–0.49) | 8.24(5.56–11.55) | 1.05(0.7–1.48) | 9.03(6.17–12.45) | 204.06 | 0.55(0.47–0.62) |
| Zambia | 0.21(0.14–0.29) | 8.42(5.74–11.78) | 0.5(0.34–0.7) | 8.8(5.94–12.04) | 142.64 | 0.14(0.08–0.2) |
| Zimbabwe | 0.3(0.21–0.43) | 8.98(6.29–12.35) | 0.51(0.34–0.72) | 9.01(6.23–12.22) | 68.28 | −0.16(−0.28–−0.05) |

YLDs: years lived with disability; EAPC: estimated annual percentage change; ASR, age-standardized rate; CI, confidence interval; UI: uncertainty interval.
